# Adjunctive Therapies in Periodontitis: Current Concepts and the Future

**DOI:** 10.1111/jre.70060

**Published:** 2026-01-20

**Authors:** Rafael Scaf de Molon, Joao Paulo Steffens, Erica Dorigatti de Avila, Wim Teughels, Thomas E. Van Dyke

**Affiliations:** ^1^ Department of Diagnostic and Surgery, School of Dentistry São Paulo State University (UNESP) São Paulo Brazil; ^2^ Department of Stomatology Universidade Federal Do Paraná (UFPR) Paraná Brazil; ^3^ Department of Oral Health Sciences KU Leuven and Dentistry (Periodontology), University Hospitals Leuven Leuven Belgium; ^4^ Department of Periodontology, Faculty of Dentistry Chulalongkorn University Bangkok Thailand; ^5^ Department of Restorative Dentistry, Faculty of Dentistry University Malaya Kuala Lumpur Malaysia; ^6^ Center for Clinical and Translational Research ADA Forsyth Institute Somerville Massachusetts USA; ^7^ Department of Oral Medicine Infection and Immunity, Faculty of Medicine Harvard University Cambridge Massachusetts USA

**Keywords:** adjunctive therapies, host modulation, inflammation resolution, natural compounds, periodontitis, photodynamic therapy, probiotics, translational research

## Abstract

Periodontitis is a chronic, host‐mediated inflammatory disease in which microbial dysbiosis and dysregulated immune responses drive the destruction of tooth‐supporting tissues. Although conventional therapy remains centered on mechanical biofilm control, persistent inflammation and alveolar bone loss in susceptible individuals underscore the need for adjunctive strategies. Translating preclinical discoveries into predictable clinical outcomes, however, remains a major challenge in periodontal research. This narrative review integrates two interrelated themes, translational research methodology and adjunctive therapeutic innovation, to critically examine how preclinical findings can be more effectively bridged to clinical practice in periodontitis management. Evidence was synthesized from experimental, translational, and clinical studies retrieved from PubMed, Scopus, and Web of Science up to September 2025. Emphasis was placed on mechanistic insights, model validity, and translational feasibility across host‐modulatory, natural, probiotic, and device‐based adjuncts. Animal models remain indispensable for mechanistic understanding and therapeutic testing but face biological and methodological limitations that hinder direct extrapolation to humans. Interspecies differences, short disease kinetics, and non‐standardized endpoints constrain translational predictability. Addressing these gaps requires refined modeling, standardized outcomes, and integration of systemic risk factors. Within this methodological framework, several adjunctive modalities, including specialized pro‐resolving mediators, probiotics, natural compounds such as curcumin and resveratrol, and device‐based therapies like antimicrobial photodynamic therapy demonstrate promising anti‐inflammatory, osteoimmunomodulatory, and regenerative effects. Emerging translational tools such as bioresponsive drug delivery systems, nanocarriers, 3D‐printed scaffolds, and AI‐driven precision periodontics may further enhance clinical relevance and patient‐specific targeting. Advancing adjunctive periodontal therapy demands harmonized translational models, bioresponsive delivery platforms, and precision diagnostic tools that bridge preclinical efficacy with real‐world outcomes. By aligning methodological rigor with therapeutic innovation, translational research can accelerate the safe and effective clinical integration of next‐generation adjunctive treatments in periodontitis.

## Graphical abstract

This graphical abstract summarizes the translational pathway from preclinical research to clinical implementation of adjunctive therapies for periodontitis, highlighting key barriers, innovative therapeutic strategies, and emerging tools that aim to bridge experimental discoveries with patient care.

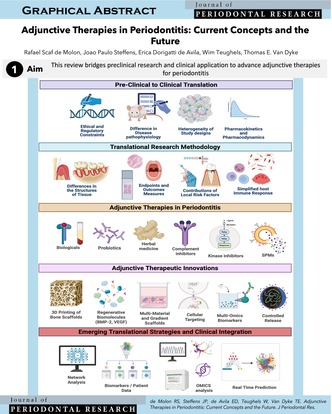

## 1 Introduction

### 1.1 Background and Summary of Essential Concepts

**FIGURE 1:**
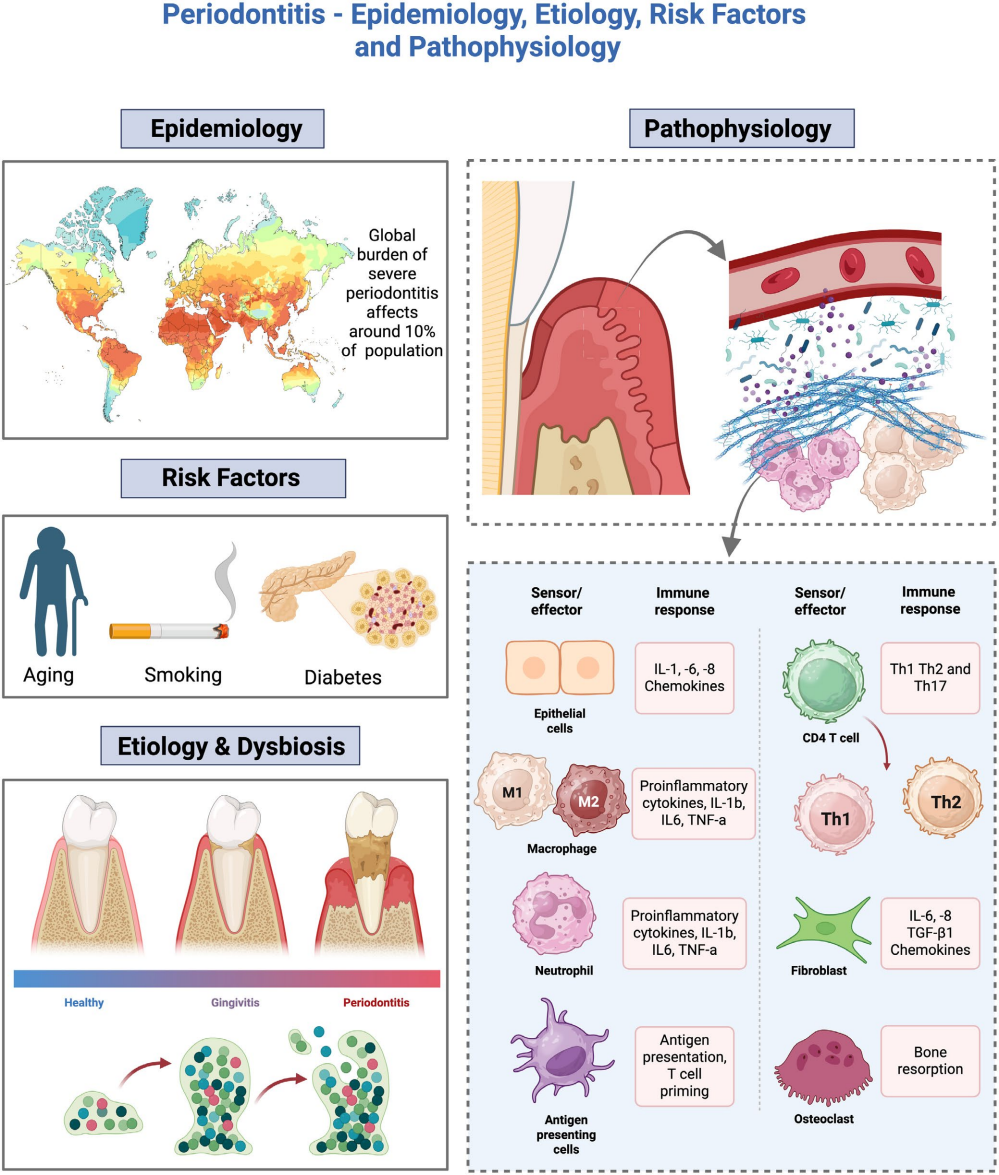
Schematic representation of periodontitis: Epidemiology, risk factors, etiology‐dysbiosis, and pathophysiology. Severe periodontitis affects approximately 10% of the global population and is strongly associated with aging, smoking, diabetes, and other systemic or lifestyle risk factors. Disease reflects the interplay between dysbiotic biofilms and host immune‐inflammatory responses, including neutrophils, macrophages, lymphocytes (Th1, Th2, Th17), antigen presenting cells, and proinflammatory cytokines (e.g., IL‐1β, IL‐6, TNF‐α), culminating in osteoclast activation and alveolar bone loss.

Periodontitis is among the most common chronic inflammatory conditions worldwide and a leading cause of tooth loss, with severe forms affecting roughly 1 in 10 adults and contributing substantially to the 3.5 billion people living with untreated oral diseases globally [1, 2, 3]. Its burden rises with age and concentrates in socioeconomically disadvantaged groups, amplifying oral–systemic health inequities [4, 5, 6]. It is characterized by the progressive destruction of the tooth‐supporting structures, including the periodontal ligament, connective tissue attachment, and alveolar bone. This multifactorial disease impairs oral function, reduces quality of life, and places a heavy economic burden on healthcare systems. Epidemiological studies have consistently identified major risk factors, including aging, tobacco use, poor oral hygiene and plaque accumulation, and uncontrolled diabetes mellitus. Diabetes shows a bidirectional link, as hyperglycemia worsens periodontal breakdown, while periodontitis further impairs glycemic control. Other contributing factors include obesity, psychosocial stress, and low socioeconomic status, all of which increase susceptibility and disease severity [7, 8, 9]. In addition, recent evidence points to the influence of genetic and epigenetic predispositions, as well as systemic conditions such as metabolic syndrome and immune disorders, which can shape disease onset, progression, and response to therapy (Figure 1) [7, 8, 9].

Beyond these risk determinants, contemporary models of pathogenesis have emphasized that the host inflammatory and immune response is central to disease progression [9, 10, 11]. While microbial colonization of the tooth surface initiates gingival inflammation, it is now clear that the persistence and dysregulation of the host response, not the presence of keystone species alone, drive the transition from gingivitis to periodontitis [12, 13, 14, 15]. Excessive or non‐resolving inflammation alters the gingival microenvironment, enriching it with hemoglobin, plasma proteins, and collagen peptides that selectively promote the outgrowth of pathobionts such as *Porphyromonas gingivalis* , *Tannerella forsythia* , and *Treponema denticola* . Longitudinal and experimental studies show that dysbiosis typically follows, rather than precedes, the onset of inflammation, indicating that host immune dysfunction is the critical determinant of microbial shifts [16, 17, 18, 19, 20]. This paradigm reframes periodontitis as a disease with microbial etiology but inflammatory pathogenesis: while bacteria initiate the process in all individuals, it is the host's inability to resolve inflammation that sustains dysbiosis, perpetuates tissue destruction, and explains why only a subset of individuals with similar microbial exposures develop severe disease [9]. The pathogenesis reflects a self‐sustaining feed‐forward loop between dysbiotic biofilms and a dysregulated host inflammatory response: innate and adaptive immune cells produce cytokines [Interleukin (IL)‐1β, tumor necrosis factor alpha (TNF‐α), IL‐6], chemokines, eicosanoids, and matrix metalloproteinases (MMP) that damage connective tissues [10]; osteoimmunologic crosstalk (RANKL/OPG imbalance, including osteocyte‐derived RANKL) drives osteoclastogenesis and alveolar bone resorption; and complement–TLR crosstalk and Th17/Treg imbalance perpetuate chronicity and systemic spill‐over [10, 13, 21, 22, 23, 24].

An essential dimension of this pathogenic loop is the failure to resolve inflammation, which is increasingly recognized as a hallmark of periodontitis [9, 11, 25]. Under physiological conditions, inflammation is terminated through specialized pro‐resolving mediators (SPMs), including lipoxins, resolvins, protectins, and maresins, which actively restore tissue homeostasis rather than simply suppressing the immune response [9, 26, 27, 28, 29, 30]. In periodontitis, however, these resolution pathways are impaired, resulting in chronic, non‐resolving inflammation that fuels tissue destruction. Instead of completing the transition from initiation to resolution, inflammatory circuits remain locked in a feed‐forward state, sustaining neutrophil infiltration, excessive production of prostaglandin E2 (PGE2) and leukotriene B4 (LTB4), and impaired phagocytic clearance of apoptotic cells. This failure in resolution not only perpetuates connective tissue and bone breakdown but also creates a nutrient‐rich, inflamed environment that promotes the persistence of dysbiosis. Experimental models have shown that restoring resolution pathways, for example, through the topical application of resolvin E1, E2 or lipoxin analogs, can reverse dysbiosis, suppress osteoclastogenesis, and even regenerate periodontal tissues without direct antimicrobial activity [26, 27, 31, 32, 33, 34]. Thus, the inability to actively resolve inflammation is central to periodontitis pathogenesis, linking microbial dysbiosis, immune dysregulation, and alveolar bone resorption into a self‐perpetuating disease process [9].

### 1.2 Translational Gap Between Pre‐Clinical Models and Clinical Outcomes

**FIGURE 2:**
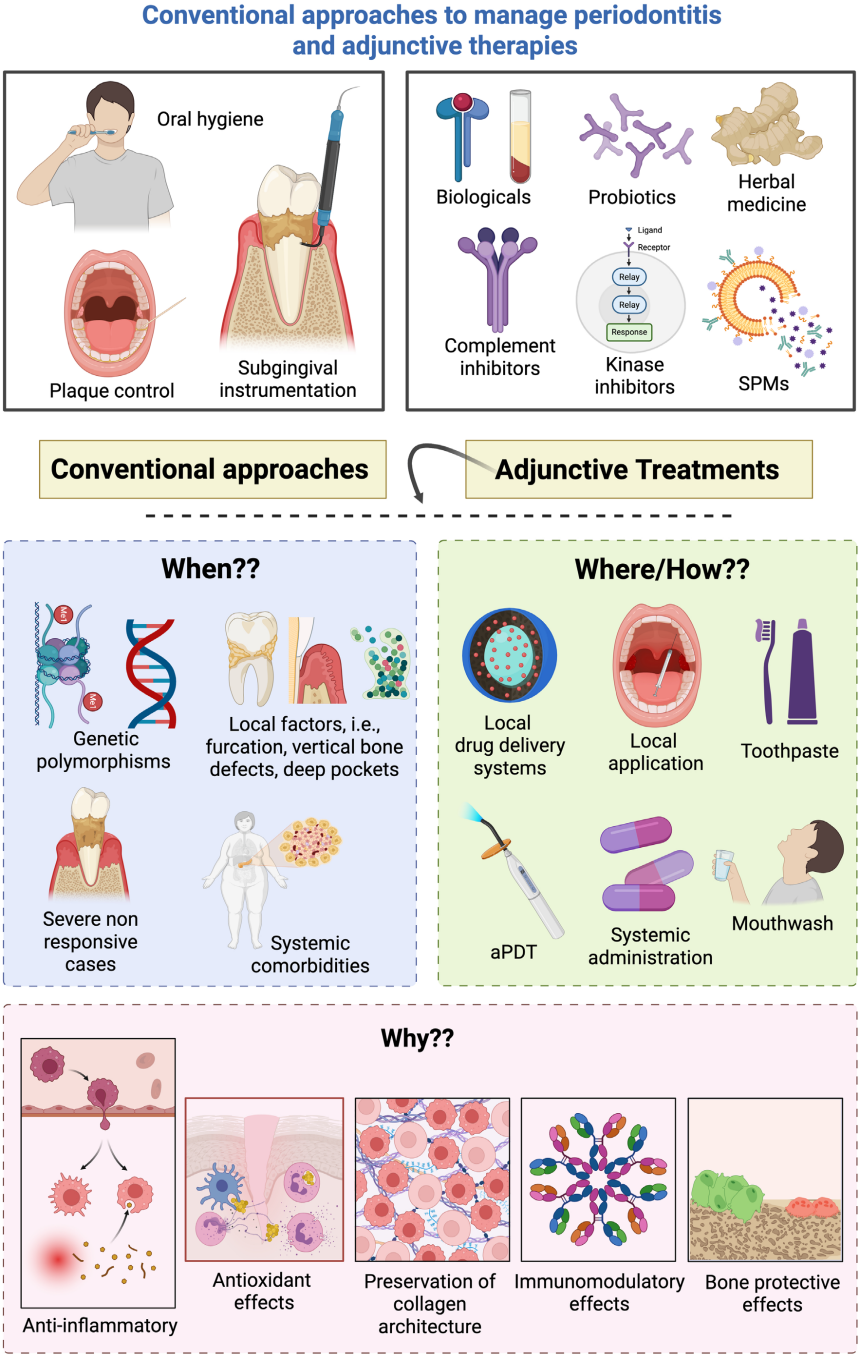
A comprehensive conceptual framework for the management of periodontitis. This diagram outlines the spectrum of strategies, from conventional foundational therapies to advanced adjunctive treatments. Conventional approaches (left) form the cornerstone of management, focusing on mechanical plaque control, subgingival instrumentation, and oral hygiene. Adjunctive therapies (top) encompass a range of behavioral, biological and pharmacological agents, such as probiotics, herbal medicines, complement inhibitors, kinase inhibitors, and SPMs, which aim to modulate the host immune response and resolve inflammation. The framework further delineates the clinical decision‐making process: “When?” Indications for adjunctive strategies include genetic polymorphisms, local anatomical factors (e.g., furcations, vertical bone defects, deep pockets), severe non‐responsive cases, and systemic comorbidities; “Where/How?” Application methods are specified, ranging from lifestyle interventions to local drug delivery systems (toothpaste, mouthwash, local application) and systemic administration and laser therapy; “Why?” The underlying therapeutic objectives are highlighted, including anti‐inflammatory, antioxidant, immunomodulatory, and bone‐protective effects, as well as the preservation of collagen architecture. While these adjunctive treatments offer promising mechanisms to enhance clinical outcomes by targeting the host's inflammatory response, their translation into routine clinical practice remains limited by heterogeneity in study models, trial designs and uncertainty, and regulatory barriers.

Conventional management of periodontitis remains risk‐ and stage‐based, with behavior modification (smoking cessation, diabetes control), meticulous plaque control, and subgingival instrumentation as the cornerstones of therapy (Figure 2). These approaches primarily suppress the microbial biofilm and mitigate the associated inflammatory burden but do not always achieve complete resolution of the disease [35, 36]. Consequently, adjunctive and host‐modulatory strategies have emerged as promising avenues to enhance treatment outcomes, particularly in challenging clinical scenarios involving local (such as deep periodontal pockets, furcation involvement, or sites showing incomplete response to conventional therapy) or systemic challenges (e.g., smokers and individuals with diabetes mellitus).

A wide array of adjunctive approaches has been explored in both pre‐clinical and clinical settings, ranging from locally delivered antimicrobials and systemic antibiotics to SPMs (e.g., lipoxins, resolvin E1, and resolvin D2) [9, 31, 32, 34, 37, 38, 39], which restore resolution pathways and mitigate bone loss. Clinical translation is also emerging: a phase I–IIa trial demonstrated reductions in gingival inflammation and probing pocket depth through complement C3 inhibition (e.g., AMY‐101) [40, 41]. In parallel, nutraceuticals such as vitamin D [34] and probiotics have shown potential by reshaping the oral microbiome, decreasing pathogenic species such as *P. gingivalis* , and modulating host immune responses to attenuate inflammation in pre‐clinical studies and clinical trials [42, 43, 44, 45, 46, 47, 48, 49]. Moreover, small molecules targeting specific signaling pathways [10, 25], and natural products (curcumin [50, 51, 52], tanshinone [53, 54], resveratrol [55], Csin‐CPI2 [56]) targeting specific inflammatory axes [10, 25, 37, 57, 58] play a role in disease management. Other than exogenous products, lifestyle interventions (such as diet modification and exercise prescription) are also biologically plausible to act as adjunctive therapies and should be further explored in pre‐clinical models, particularly due to bias derived from behavioral modification in such clinical trials [59] (Figure 2). Therefore, not only does translational science involve potential clinical application of pre‐clinical models, but it also demands proof‐of‐concept of clinical experiments where biases cannot be adequately controlled.

However, despite encouraging pre‐clinical evidence, translating these findings into predictable clinical benefits remains a significant challenge. Differences in disease chronicity, environmental exposures, immune phenotypes, and comorbid conditions, such as diabetes and smoking, often limit the reproducibility of pre‐clinical successes in human trials. Furthermore, the heterogeneity of periodontitis, encompassing variable microbial communities and host susceptibilities, complicates the assessment of adjunctive efficacy. Bridging this translational gap requires robust experimental designs, standardized outcome measures, and interdisciplinary collaboration to ensure that promising biologics and biomaterials can be effectively and safely integrated into clinical practice [51, 53, 55, 56, 60, 61, 62].

### 1.3 Rationale and Aims of This Narrative Review

While the pipeline of treatments for periodontitis is encouraging, most are not available in the clinic as further research is needed to complete translation. This final step, which is fraught with roadblocks, is the subject of this review, which focuses on how the strengths and limitations of animal and translational models, especially their ability to recapitulate human dysbiosis–inflammation dynamics and osteoimmunology, either enable or impede clinical implementation of adjunctive, inflammation‐modulating therapies in periodontitis. Accordingly, this work adopts a dual approach. Firstly, it explores the methodological and biological constraints that contribute to the translational gap between experimental discoveries and clinical implementation in periodontitis. Secondly, it critically reviews emerging adjunctive therapies, including host modulators, natural compounds, probiotics, and device‐based interventions, within this translational framework. By merging these perspectives, this review highlights how advances in experimental design, delivery systems, and biomarker validation can accelerate the safe and effective adoption of novel therapies in clinical practice.

For this narrative review, the literature was identified through targeted searches in PubMed, Scopus, and Web of Science databases up to October 2025, using combinations of keywords such as *periodontitis*, *animal models*, *host modulation*, *inflammation resolution*, *translational research*, and *adjunctive therapies*. Additional studies were included based on their relevance, methodological quality, and contribution to understanding translational mechanisms or therapeutic innovation in periodontology. By integrating evidence from experimental, translational, and clinical studies, this review aims to highlight conceptual and methodological strategies to bridge the gap between preclinical advances and clinical implementation.

The adjunctive interventions discussed in this review were selected based on their novelty translational relevance and the availability of both preclinical and clinical data supporting their mechanistic rationale or therapeutic potential. Priority was given to therapies that exemplify key challenges in bridging preclinical discovery to clinical application, namely, host‐modulatory agents, natural products, probiotics, small‐molecule inhibitors, and device‐based approaches such as antimicrobial photodynamic therapy. Other adjunctive modalities, including behavioral modification, systemic antimicrobials, ozone therapy, low‐dose doxycycline, and platelet‐rich preparations, were not examined in detail because their mechanisms, regulatory pathways, or translational barriers differ substantially from the scope of this review. Nonetheless, these approaches remain important complementary strategies in contemporary periodontal therapy and warrant dedicated discussion elsewhere.

## 2 Methodological Foundations: The Role and Advantages of Animal Models

Animal models are crucial in translational clinical research, helping bridge the gap between basic science discoveries and their application in human medicine. Rodents, particularly rats and mice, are commonly used as animal models to study periodontitis. Their periodontal anatomy, especially in the molar region, and the structure of the periodontium, including the gingiva and alveolar bone, share similarities with humans, making them suitable for research. Additionally, rodents are relatively small, have short lifespans, allow for genetic manipulation, are readily available, and are relatively inexpensive, which, in turn, allows for adequate sample sizes and statistical power [11, 63, 64, 65, 66].

Animal models have played an essential role in advancing our understanding of periodontitis pathogenesis and in developing preventive and therapeutic strategies [10, 64, 66, 67, 68]. Although no model perfectly reproduces the human condition, carefully designed preclinical systems offer unique opportunities to investigate mechanistic pathways, test hypotheses, and evaluate novel interventions under controlled conditions that are not feasible in humans for ethical, practical, or technical reasons [63, 64, 65, 66, 68]. These models have been instrumental in elucidating the complex interplay between microbial dysbiosis and host immunity that drives periodontal destruction [63, 64, 65, 67, 68, 69]. By allowing controlled manipulation of the microbial challenge, whether through the introduction of specific pathogens such as *P. gingivalis* or *Aggregatibacter actinomycetemcomitans* , or through ligature placement and/or contamination (oral inoculation), animal studies can establish causal relationships that complement the associative data derived from human observational research [63, 64, 65, 67, 68, 69].

Moreover, genetically modified and humanized rodent models enable targeted interrogation of cytokines, signaling molecules, and immune cell subsets, providing valuable insights into how specific components of the immune system contribute to bone resorption and tissue breakdown [10, 70]. The ability to incorporate systemic modifiers such as diabetes, obesity, or aging into these models further enhances their relevance, allowing researchers to explore how systemic inflammation, metabolic dysregulation, or immunosenescence influences disease susceptibility and the capacity for repair [71, 72, 73, 74, 75].

Another advantage of animal models lies in their capacity to shed light on the temporal dynamics of disease progression [10, 63, 67, 68, 69]. The controlled induction of periodontitis makes it possible to capture early molecular and histological events that precede overt clinical destruction, providing opportunities to identify potential biomarkers for early detection. This temporal control also facilitates the evaluation of preventive strategies, as interventions can be administered before or during the initial stages of disease, something that is rarely possible in human studies. In addition, certain designs allow the study of disease recurrence and maintenance therapy, offering important insights into long‐term management strategies. The controlled nature of these models enables precise evaluation of prophylactic efficacy, recurrence rates, and the effects of supportive periodontal therapy on preventing reactivation [75, 76, 77, 78, 79, 80].

Animal models are useful for preclinical evaluation of therapeutic interventions, bridging the gap between in vitro assays and human clinical trials [25, 61, 62]. Host‐modulatory drugs, anti‐inflammatory agents, antiresorptive medications, and regenerative strategies such as bone grafts, barrier membranes, growth factors, and stem‐cell–based therapies have been assessed in vivo to determine their effects on inflammation, bone resorption, and tissue regeneration [10, 25, 61, 62]. Similarly, local drug delivery systems, including chitosan‐based hydrogels, biodegradable microspheres, and nanoparticle carriers, have been tested in animal models to assess retention, release kinetics, bioactivity, and biocompatibility under the dynamic conditions of the oral cavity [55, 81, 82]. These studies enable dose optimization, pharmacokinetic profiling, and safety assessments before candidate therapies progress to human trials, thus reducing risk and increasing the likelihood of clinical success.

From an ethical and practical standpoint, animal studies allow for experimental designs that would be impossible in humans. On the other hand, the use of animals in research is being discouraged worldwide, particularly when alternative methods are available, according to the 3R's principles (replacement, reduction and refinement) [83]. Deliberate induction of periodontitis and/or other systemic conditions, invasive tissue sampling at multiple time points, and testing of high‐risk experimental agents cannot be justified in patient populations, yet they are critical for understanding disease mechanisms. The diversity of available animal systems, from rodents suited for genetic manipulation and mechanistic exploration to large animal models such as dogs and non‐human primates that offer closer anatomical, microbial, and immunological similarities to humans, provides a flexible framework in which early‐stage hypotheses can be refined before being tested in more clinically relevant settings. Besides, animal models also help establish biological plausibility, especially for questions confounded by human behavioral factors (e.g., compliance or the Hawthorne effect in clinical trials). Therefore, translational science focuses not only on how findings on preclinical models may extrapolate to humans but also on how clinical observations in humans may assist in developing relevant models for understanding a given condition [67, 69, 84, 85].

Historically, animal models have been instrumental in shaping foundational concepts in periodontology [86]. They have facilitated the recognition of bacterial biofilms as key etiological agents, supported the development and validation of host modulation therapies, and enabled the refinement of regenerative techniques that are now standard in clinical practice [87]. Moreover, they have provided the preclinical efficacy and safety data required for regulatory approval of several adjunctive therapies and local antimicrobials. Despite inherent limitations that demand careful experimental design and cautious interpretation, animal models remain indispensable for advancing our understanding of disease biology, identifying biomarkers, optimizing drug delivery systems, and reducing the risks associated with clinical translation [88, 89]. When integrated with in vitro studies and human trials, they form a crucial link in the translational research continuum, fostering clinically relevant innovations for periodontitis prevention and treatment. Nevertheless, significant challenges persist, particularly in replicating the complex mechanisms underlying disease pathogenesis and treatment response in humans, which will be the focus of the next section.

## 3 Methodological Constraints Limiting Translation From Pre‐Clinical to Clinical Research

**FIGURE 3:**
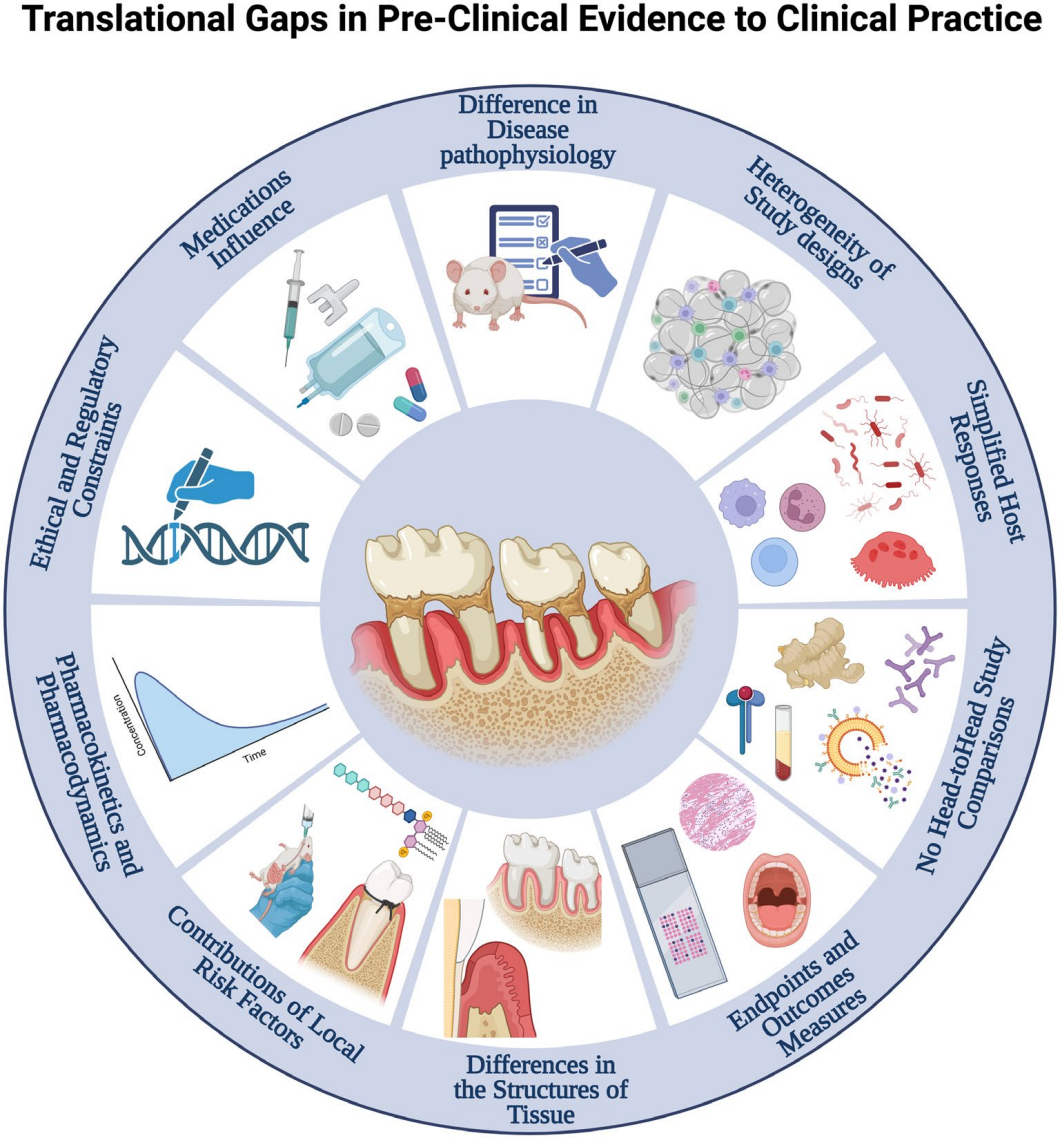
Translational gaps from preclinical evidence to clinical practice in periodontitis research. This schematic illustrates ten key barriers limiting the direct translation of preclinical findings into clinical applications for periodontitis. Factors include: (1) differences in disease pathophysiology between animal models and humans; (2) heterogeneity in study design; (3) simplified host response models; (4) absence of head‐to‐head study comparisons; (5) variability in endpoints and outcome measures; (6) structural differences in periodontal tissues across species; (7) local risk factor contributions (e.g., smoking, diabetes); (8) pharmacokinetic and pharmacodynamic inconsistencies; (9) ethical and regulatory considerations; and (10) medication influences that may alter disease progression or therapeutic responses. Addressing these gaps is essential for improving the predictive value and clinical applicability of preclinical research in periodontitis.

Despite major advances in preclinical methodologies, translational predictability remains constrained by differences in host response, pharmacokinetics, and disease chronicity across species [66]. Ethical and regulatory pressures further restrict the scope of animal experimentation, particularly for chronic or high‐risk interventions. Bridging these barriers demands standardized protocols, inclusion of systemic risk factors, and prioritization of clinically meaningful outcomes. These limitations are comprehensively summarized in Figure 3 and Table 1, as well as in Sections 3.1, 3.2, 3.3 below. The next section builds on these methodological insights to explore how translational innovations can drive the development of next‐generation adjunctive therapies.

**TABLE 1:** Summary table categorizing translational barriers into thematic domains for clarity.

Barrier domain	Specific barriers	Translational implications
Pathophysiology and Biology	Differences in disease pathophysiology; structural differences in periodontal tissues across species	Animal results may not reflect human mechanisms of disease onset, progression, or repair
Study Design and Methodology	Heterogeneity in study design; absence of head‐to‐head comparisons; variability in endpoints/outcome measures	Inconsistent data prevents reliable cross‐study synthesis and weakens evidence base
Host Response Models	Simplified host immune/inflammatory responses	Fails to replicate multifactorial human disease; risk of oversimplified therapeutic effects
Risk Factors and Comorbidities	Local/systemic risk factors not fully modeled (e.g., smoking, diabetes)	Reduces ecological and clinical relevance of findings; may overlook key modifiers of treatment outcomes
Pharmacology and Therapeutics	Pharmacokinetic/pharmacodynamic inconsistencies across species; medication influences on disease or therapy	Limits predictive value for dosing, safety, and efficacy in human trials
Ethical and Regulatory	Ethical restrictions and evolving regulations regarding animal use	Constrains experimental scope; may slow approval and increase reliance on alternative models

### 3.1 Biological and Pathophysiologic Constraints

The intricate host–microbiome interactions (ecological and immunological insights) during periodontitis pathogenesis [2, 90, 91, 92, 93, 94, 95, 96, 97] underscore the limitations of current animal models. Common preclinical approaches, such as oral gavage or inoculation with human pathogens ( *P. gingivalis* , *A. actinomycetemcomitans* ), assume that introducing exogenous organisms into rodents will recapitulate human disease [63, 64, 65, 66, 67, 68]. However, rodents neither naturally harbor these organisms nor share the same microbiota composition, immune complexity, or ecological dynamics as humans [98, 99]. As a result, human pathogens often exhibit non‐physiological colonization patterns, interact atypically with the murine immune system, and fail to generate the chronic, episodic, and multifactorial disease course seen clinically [98].

This recognition has driven increasing use of ligature‐induced models, in which mechanical disruption of the native murine biofilm provokes local inflammation, nutrient shifts, and overgrowth of endogenous pathobionts [66, 100]. These models better reproduce the ecological imbalance central to disease pathogenesis because dysbiosis emerges within the host's indigenous microbiota rather than from artificial inoculation with foreign organisms. Nevertheless, ligature models typically generate acute inflammation with rapid bone loss, contrasting sharply with the prolonged, heterogeneous, and host‐modulated disease trajectory observed in humans [66, 100].

Host‐related factors further complicate model interpretation. Genetic background, epigenetic regulation, age, diet, and housing conditions all influence immune tone, microbial composition, and bone biology, thereby shaping susceptibility and response to perturbations [98, 101]. Moreover, systemic comorbidities highly relevant to human periodontitis, such as diabetes, obesity, smoking, or age‐related immune senescence, are absent from most standard models despite their known effects on inflammatory networks, bone metabolism, and treatment outcomes [102]. Incorporating these variables is technically feasible but substantially increases experimental complexity, cost, and ethical burden.

Finally, species‐specific immunological differences constrain direct extrapolation. Murine models differ from humans in neutrophil recruitment kinetics, cytokine and chemokine networks, bone turnover rates, and adaptive immune responses [98]. Such divergences affect not only disease susceptibility and progression but also the pharmacodynamics and pharmacokinetics of candidate therapies, thereby limiting translational relevance.

### 3.2 Challenges in Modeling Treatment and Repair

While the above limitations apply primarily to disease induction, additional constraints emerge when animal models are used to evaluate therapeutic interventions or healing responses. The ligature‐induced model remains the most widely used system because it produces reproducible bone loss within a defined time frame [66]. Yet the removal of the ligature, often employed as a surrogate for therapeutic intervention, triggers rapid spontaneous resolution of inflammation and partial bone stabilization even without any additional intervention [103, 104, 105, 106, 107]. This self‐limited repair phase can mask or attenuate the incremental benefits of experimental therapies, particularly those targeting inflammatory pathways rather than bacterial burden. This has been overcome in some models by leaving ligatures in place during the therapy phase of the experiment [31], although the clinical extrapolation of those can be questioned.

Temporal dynamics introduce further complexity. The progression from disease induction to peak inflammation and subsequent resolution in rodents unfolds over days to weeks [63, 66, 67, 108], whereas human periodontitis develops over months to years. Consequently, there is often a mismatch between model kinetics and therapeutic pharmacodynamics: if an intervention is administered after inflammation has already begun to subside spontaneously, its efficacy may be underestimated. Conversely, interventions delivered during hyperacute inflammatory phases may overestimate efficacy relative to chronic human disease. Standardizing baseline disease severity across experimental animals is challenging because ligature material, placement technique, duration, and host susceptibility all influence the magnitude and timing of bone loss [66, 100, 109]. Moreover, the time‐dependent effects of adjunctive therapies, such as pharmacological agents, necessitate careful methodological consideration. For instance, should the pharmacological treatment begin before or concurrently with the induction of periodontal repair? This decision is crucial, as it may affect baseline disease severity and confound the interpretation of true adjunctive effects. Conversely, if the drug's pharmacokinetics and pharmacodynamics require a longer period to exert biological activity, studies with limited observation periods may misleadingly suggest a lack of efficacy on periodontal repair.

Reproducing mechanical debridement, the clinical cornerstone of periodontal therapy, poses additional obstacles. Subgingival instrumentation in small animals is technically demanding, difficult to standardize, and may provide minimal to no additional benefit beyond ligature removal alone [55, 60, 107, 110]. Furthermore, anatomical differences such as fenestrations in murine molars (e.g., buccal vs. palatal surfaces), variations in bone density, root anatomy, and oral microbiome composition influence disease dynamics and treatment responses further restricting translational applicability and increasing variability [66, 100]. Likewise, the combination of local risk factors, including biomechanical forces, diet, and oral hygiene practices, differs markedly between controlled laboratory settings and real‐world human conditions, potentially masking or exaggerating therapeutic effects in animal studies (Figure 3).

### 3.3 Methodological and Translational Gaps

Beyond biological and technical issues, methodological choices in study design and endpoint selection contribute to the translational gap between preclinical findings and clinical practice. Most animal studies emphasize surrogate endpoints, micro‐CT bone measurements, histologic scoring, or molecular markers of inflammation and bone metabolism, because they offer high‐resolution mechanistic insights [8, 10, 13, 25, 66, 98, 111]. However, these outcomes rarely align with clinically meaningful endpoints such as probing depth reduction, clinical attachment gain, tooth mobility stabilization, long‐term tooth retention, or patient‐reported measures like pain and quality of life. As a result, therapies showing substantial histologic or molecular improvements in rodents may fail to deliver sustained clinical benefits in human trials. Besides, analytical approaches across laboratories hinder reproducibility and make cross‐study comparisons difficult. Compounding this issue is the scarcity of head‐to‐head comparisons between adjunctive therapies, leaving clinicians with limited guidance on relative efficacy when multiple interventions appear promising in isolation (Figure 3).

Preclinical models inherently fail to reproduce the behavioral, psychosocial, and environmental determinants that modulate disease progression and therapeutic outcomes in humans. Factors such as oral hygiene habits, stress, diet, systemic health, and socioeconomic status have a profound impact on the host response and treatment success, yet remain outside the scope of controlled laboratory settings. This disconnect contributes to the persistent gap between preclinical efficacy and clinical effectiveness, underscoring the need for translational frameworks that integrate behavioral and social determinants of health into periodontal research and therapy evaluation.

Pharmacokinetic and pharmacodynamic differences further limit extrapolation and add another layer of complexity. Small animals metabolize and distribute drugs differently from humans, affecting local drug concentrations, systemic exposure, and dosing regimens. Consequently, effective doses in rodents often over‐ or underestimate therapeutic windows in human patients, particularly for biologics or small molecules with narrow safety margins, thus complicating dose extrapolation and efficacy predictions for human application (Figure 3). Adjuncts applied locally in preclinical pockets may achieve concentrations or retention times that are not reproducible in the clinical setting due to differences in pocket architecture, salivary flow, and mechanical disruption, thus inflating perceived efficacy in the model relative to expectable human performance [111].

Systemic administration of anti‐inflammatory drugs, osteoimmunomodulatory compounds, or host‐modulating biologics exposes the entire body to pharmacologic effects intended for a disease that is anatomically localized. This approach creates an unfavorable risk–benefit balance, as the potential systemic adverse effects, including gastrointestinal, hepatic, cardiovascular, or immunologic complications, often outweigh the benefits of treating a localized periodontal lesion. In addition, the systemic route frequently fails to achieve sufficiently high concentrations at the periodontal site due to pharmacokinetic limitations, resulting in subtherapeutic exposure within the inflamed tissue microenvironment [10, 25, 61, 62].

Finally, ethical and regulatory constraints shape translation. Periodontitis is a chronic, non–life‐threatening condition; thus, experimental interventions carrying significant systemic risks face stringent safety barriers before entering early‐phase human trials. This cautious approach contrasts with preclinical studies, where high‐risk therapies may be tested under controlled conditions without equivalent regulatory hurdles.

In summary, bridging the translational gap requires integrated and methodologically sound strategies. These include the development of standardized protocols for disease induction and therapeutic evaluation, the incorporation of host susceptibility factors such as aging, metabolic disorders, or bone diseases, and the longitudinal profiling of immune and microbial dynamics. Equal emphasis should be placed on prioritizing clinically relevant outcomes that reflect real‐world therapeutic benefits. Furthermore, direct head‐to‐head comparisons of promising adjunctive therapies, supported by harmonized measurement frameworks across laboratories, are essential to ensure reproducibility and comparability of findings. Ultimately, triangulating evidence from in vitro models, animal studies, and human clinical research provides the most reliable pathway to accelerate the development of effective adjunctive interventions for periodontitis (Table 2). Building upon the methodological and technological principles discussed earlier, the next section critically examines key categories of adjunctive therapies, ranging from host‐modulatory agents to natural products and device‐based approaches highlighting how each addresses, or remains limited by, these same translational constrictions.

**TABLE 2:** Strengths and limitations of animal models in periodontal research.

Aspect	Strengths/advantages	Limitations/constraints
Anatomical and Biological Relevance	Rodents and large animals (dogs, primates) share key periodontal structures with humans, enabling investigation of microbial dysbiosis and host–immune interactions. Large animals provide closer anatomical and microbial similarity to humans	Inter‐species differences in immune responses, tissue repair, and microbiota; human pathogens often colonize non‐physiologically in rodents
Experimental Control	Allow deliberate induction of periodontitis (e.g., ligature or inoculation) and controlled manipulation of systemic risk factors such as diabetes, obesity, and aging	Do not fully reproduce the chronic and multifactorial course of human periodontitis; host–microbiome interactions remain simplified
Mechanistic Insights	Enable causal analysis of cytokine signaling, immune cell subsets, and molecular pathways using genetically modified or humanized models	Cannot fully recapitulate the ecological and immunological complexity of human disease networks
Therapeutic Testing	Permit in vivo evaluation of host‐modulatory, antiresorptive, and regenerative therapies under dynamic oral conditions, supporting dose optimization and pharmacokinetic studies	Pharmacokinetics and pharmacodynamics vary across species; outcome measures are not standardized between studies
Ethical/Practical Flexibility	Allow invasive sampling, repeated biopsies, and high‐risk interventions not feasible in human subjects	Increasing regulatory/ethical pressure to reduce animal use; limitations in extrapolating high‐risk interventions. Increasing discouragement in the US/EU for animal efficacy testing further limits their role
Historical Contribution	Provided foundational insights into biofilm etiology, host modulation, and periodontal regeneration; contributed data supporting regulatory approval of adjunctive treatments	Persistent translational gaps remain due to heterogeneous study designs, lack of direct model comparisons, and limited external validity
Temporal Dynamics and Longitudinal Insights	Controlled induction enables study of early molecular and histological events, preventive interventions, recurrence, and maintenance phases in longitudinal settings	Experimental disease progression is typically more acute and less multifactorial than the chronic, episodic pattern seen in humans

## 4 Adjunctive Therapies for the Treatment of Periodontitis

The growing understanding that periodontitis is primarily a host‐mediated inflammatory disease rather than a purely infection‐driven condition has redefined therapeutic priorities. Beyond conventional mechanical debridement, contemporary research increasingly focuses on adjunctive interventions capable of modulating the host immune response, restoring microbial homeostasis, and promoting tissue regeneration. These adjunctive approaches encompass a wide range of biological, pharmacological, and device‐based modalities designed to complement standard care. This section provides a comprehensive overview of the principal classes of adjunctive therapies highlighting their mechanistic rationale, preclinical evidence, clinical applicability, and translational challenges.

### 4.1 Localized Host Modulatory Therapies

A promising and rapidly expanding class of agents for localized delivery in periodontitis is the SPMs. These are endogenous bioactive lipids, including lipoxins, resolvins, protectins, and maresins, derived from omega‐6 and omega‐3 fatty acids that actively orchestrate the resolution of inflammation rather than suppressing it [27, 29, 112, 113]. SPMs are biosynthesized from arachidonic acid, EPA and DHA that actively drive the resolution of inflammation and return tissues to homeostasis. SPMs act as receptor agonists at specific G‐protein coupled receptors (e.g., ALX/FPR2 for lipoxin A_4_ and RvD1, ChemR23/CMKLR1 for RvE1, and GPR32 for RvD1) to reprogram leukocyte responses and reduce pro‐inflammatory cytokine production while enhancing clearance of apoptotic cells and microbial debris, a mechanism that differs fundamentally from cytokine blockade or antimicrobial killing [114, 115, 116]. Since SPMs are receptor agonists, retention of the compounds locally for long periods of time is not required as it would be for enzyme inhibitors or receptor antagonists.

Preclinical studies demonstrate that SPMs not only attenuate inflammatory bone loss but also exhibit osteogenic and regenerative properties, enhancing periodontal ligament cell proliferation, osteoblast differentiation, and new bone formation at defect sites [26, 31]. Importantly, SPMs achieve these effects without the systemic side effects typically associated with immunosuppressive or anti‐resorptive therapies because they do not inhibit immune responses but instead promote the natural resolution phase of inflammation [28, 29]. SPMs have emerged as one of the most promising classes to overcome several adjunctive barriers. Acting at picogram–nanogram concentrations via receptor‐mediated pathways, SPMs can induce durable pro‐resolving and regenerative responses without requiring prolonged high‐dose exposure [40]. Previous findings establish proof‐of‐concept that minimally invasive delivery formats can achieve both local efficacy and measurable systemic pharmacodynamics [40].

From a delivery perspective, SPMs offer several practical advantages. They are highly effective when administered locally using simple, minimally invasive platforms such as mouthwashes, gels, or biodegradable scaffolds. Their lipophilic nature facilitates tissue penetration, and local absorption ensures high bioavailability at the site of disease while minimizing systemic exposure. Recent translational efforts have shown promising results, with SPM‐containing formulations already available in certain European markets, providing a clinically feasible and safe adjunctive approach for periodontitis therapy [117, 118, 119, 120]. Taken together, SPMs represent a paradigm shift in periodontal therapy, moving from microbial eradication or cytokine inhibition toward pro‐resolving, regenerative strategies that align with the complex immuno‐regenerative biology of periodontitis.

While SPMs have emerged as a particularly promising class of host‐modulatory agents in periodontal research, they represent only one part of a much broader landscape of adjunctive therapeutic strategies under investigation [10, 25, 61, 62]. Multiple agents, including natural products, probiotics, small molecules, and device‐based adjuncts such as photodynamic therapy and laser applications, have demonstrated encouraging results in preclinical models. A comprehensive understanding of the preclinical and clinical evidence for these adjunctive therapies, along with the barriers impeding their adoption into routine practice, is essential for a balanced perspective on future directions in periodontal therapy.

### 4.2 Natural Products and Phytochemicals: Curcumin, and Resveratrol

Plant‐derived compounds have gained substantial attention due to their pleiotropic pharmacologic activities, favorable safety profiles, and low production costs [121, 122]. Among these, curcumin, and resveratrol, two naturally derived polyphenols, have shown promising effects in experimental periodontitis through anti‐inflammatory, antioxidant, immunomodulatory, and bone‐protective mechanisms, and advances in drug‐delivery systems are helping to overcome their limitations in bioavailability and retention [50, 52, 123].

Curcumin, when administered systemically or locally, reduces key pro‐inflammatory cytokines such as IL‐1β and TNF‐α, inhibits NF‐κB activation, improves the OPG/RANKL balance, reduces gingival inflammation, preserves collagen architecture, and attenuates alveolar bone loss in ligature‐induced or LPS‐stimulated models [50, 52, 123]. However, systemic administration shows lesser effects on protecting bone loss unless high doses or prolonged treatment are used [123]. To enhance local efficacy, curcumin formulations such as 2% gel applied directly into periodontal pockets or intra‐pocket dental films have been developed; this shows reduced gingival indices, probing depths, and inflammation biomarkers, with release profiles featuring initial burst followed by sustained delivery to maintain therapeutic concentrations [124, 125]. Curcumin also appears to suppress ferroptosis in periodontal tissues, via upregulating antioxidant defenses (e.g., GPX4, SLC7A11), reducing malondialdehyde, and preserving glutathione, thereby limiting lipid peroxidation and cell death in alveolar bone and gingiva [126].

Resveratrol, a polyphenolic compound abundant in red wine, grapes and berries has been shown to exert protective effects against periodontitis primarily by blocking NF‐κB activation and reducing the expression of pro‐inflammatory cytokines [127]. Resveratrol has demonstrated protective effects against alveolar bone loss in experimental periodontitis [55]. Its mechanisms of action include the attenuation of oxidative stress and inflammatory signaling pathways, particularly through activation of the Nrf2/HO‐1 axis. Additionally, resveratrol has been shown to reduce ROS generation, suppress the expression of MMPs, and modulate immune responses, collectively contributing to its anti‐inflammatory and tissue‐protective properties [128, 129]. In experimental models, resveratrol exerts anti‐inflammatory effects, suppresses osteoclast differentiation, and inhibits activation of the NLRP3 inflammasome. In diabetic mice with ligature‐induced periodontitis, it has been shown to improve glycemic control and attenuate alveolar bone loss. These effects are, at least in part, mediated through the inhibition of Toll‐like receptor 4 (TLR4) signaling and downstream pathways, including STAT3, p38 MAPK, and NF‐κB [130].

Furthermore, its systemic metabolic benefits, including improved insulin sensitivity and reduced oxidative stress, make it particularly appealing for periodontitis in patients with type 2 diabetes or metabolic syndrome [131]. However, similar to curcumin, resveratrol displays poor bioavailability due to rapid intestinal metabolism and hepatic clearance [132]. Clinical trials testing resveratrol in periodontal therapy are scarce and heterogeneous, with inconclusive evidence regarding its additive benefits beyond conventional mechanical debridement [133, 134, 135]. Innovative delivery systems such as resveratrol‐loaded nanoparticles, cyclodextrin complexes, and mucoadhesive hydrogels are being developed to circumvent pharmacokinetic limitations and provide sustained local release within periodontal pockets.

To address its poor solubility and rapid metabolism, several advanced delivery systems have been explored. For example, a recent study introduced a novel therapeutic strategy for diabetes‐related periodontitis involving a coconut oil‐based solid lipid nanoparticle (SLN) system encapsulating resveratrol (RSV) within a BDDE‐crosslinked hyaluronic acid hydrogel matrix (RSV@CLgel). This dual‐release platform enhances RSV stability and overcomes limitations associated with poor bioavailability by enabling localized, sustained drug delivery, releasing approximately 15% of RSV within the first 30 min and 63.5% over 24 h. In vitro, RSV@CLgel exhibited multi‐targeted therapeutic effects, including anti‐inflammatory, antioxidant, immunomodulatory, and bone‐regenerative activities, underscoring its potential as a biocompatible and effective intervention for the complex pathophysiology of diabetic periodontitis [129].

Nanoparticle‐based delivery systems have been shown to further enhance their immunoregulatory potential in models of both periodontitis and diabetes. Moreover, a novel chitosan‐based thermosensitive injectable self‐assembled hydrogel (TISH) co‐loaded with granulocyte‐macrophage colony‐stimulating factor (GM‐CSF) and resveratrol, termed TISH (GR), was designed to modulate immune responses and accelerate periodontal healing in the context of metabolic syndrome. In vitro studies demonstrated that TISH (GR) downregulated inflammatory signaling pathways (MAPKs and NF‐κB) in dendritic cells while promoting a tolerogenic phenotype characterized by reduced TNF‐α and IL‐6 production and elevated IL‐10 levels. In a rat model of high‐fat diet‐induced periodontitis, combining TISH (GR) with conventional subgingival instrumentation significantly decreased inflammation, limited Th17 cell infiltration, and improved periodontal tissue repair compared to scaling alone [55]. Similarly, nanoparticle formulations combining resveratrol with other agents (e.g., 20(S)‐protopanaxadiol) have also been shown to modulate host immune responses by promoting M2 macrophage polarization over M1, reducing ROS levels, and ultimately enhancing periodontal healing [136].

Overall, while both agents show considerable therapeutic potential in experimental periodontitis, local drug delivery systems (gels, films, nanoparticle/hydrogel hybrids, mucoadhesive tablets) are particularly promising for maintaining effective concentrations in the periodontal pocket, controlling release, improving drug stability, and reducing systemic side effects, as discussed in Section 5 of this article. However, several clinical trials using curcumin gels or mouth rinses as adjuncts to subgingival instrumentation report reductions in probing depth and gingival indices [137, 138, 139], but meta‐analyses highlight significant heterogeneity, small sample sizes, and short follow‐up periods [140, 141]. Consequently, no definitive clinical recommendations can yet be made, and novel delivery strategies are being investigated to overcome pharmacokinetic barriers (refer to Section 5). The continuing challenge is optimizing release kinetics, adherence to tissue, mechanical stability, and ensuring that delivery platforms can be translated into safe and effective clinical products.

Collectively, these natural products illustrate a recurrent translational paradox: despite robust mechanistic rationale and compelling preclinical efficacy, pharmacokinetic hurdles, formulation challenges, and limited high‐quality clinical data have thus far precluded their routine use in periodontitis management. Future research integrating medicinal chemistry, drug delivery science, and well‐powered randomized clinical trials is needed to unlock their full therapeutic potential.

### 4.3 Probiotics and Microbiome‐Modulating Approaches

Given the role of microbial dysbiosis in periodontitis pathogenesis, probiotics have been investigated as adjunctive therapies aiming to restore a symbiotic oral microbiome and competitively inhibit pathogenic species [42, 43, 44, 45, 46, 47]. Various *Lactobacillus* and *Bifidobacterium* strains, as well as the yeast *Saccharomyces boulardii*, have demonstrated the ability to reduce periodontal inflammation and modulate local immune responses in animal models [48, 142, 143, 144]. Proposed mechanisms include competitive exclusion of pathogens, production of antimicrobial peptides (bacteriocins), modulation of local pH, and stimulation of anti‐inflammatory cytokines such as IL‐10 [48, 142, 143, 144].

Clinical trials evaluating probiotics as adjuncts to subgingival instrumentation have reported statistically significant improvements in clinical attachment level (CAL) and bleeding on probing (BOP) compared with SRP alone [42, 43, 44, 45, 46, 47]. However, results remain heterogeneous across different formulations and study designs [145, 146, 147, 148]. For example, *Lactobacillus reuteri* administered after re‐instrumentation in periodontitis patients improved probing depth reduction and increased the percentage of shallow pockets compared with placebo, although without significant changes in subgingival pathogen counts [147]. Similarly, *Streptococcus* spp. probiotic tablets used as an adjunct to SRP in periodontitis patients failed to produce additional clinical benefits beyond placebo, except for minor effects on plaque levels and *Prevotella intermedia* counts [148]. In studies on gingivitis, *Bifidobacterium animalis* ‐supplemented yogurt reduced plaque accumulation, gingival inflammation, and IL‐1β levels after a period of suspended oral hygiene, suggesting anti‐inflammatory benefits under experimental conditions [146]. However, a *Bacillus* spp.‐containing toothpaste, mouthrinse, and toothbrush cleaner did not provide clinically relevant improvements over placebo in gingivitis management [145]. Systematic reviews and meta‐analyses emphasize the high heterogeneity in probiotic strains, dosages, delivery vehicles (lozenges, tablets, dairy products), treatment durations, and patient populations [149, 150, 151, 152]. Moreover, individual variability in host response and baseline microbiome composition likely influences outcomes, while most studies employ short‐term follow‐up, leaving the durability of benefits uncertain.

More recent trials also illustrate this complexity. Lu et al. [153] reported that *L. reuteri* alleviated periodontitis by suppressing endoplasmic reticulum stress and inflammatory pathways, leading to significant reductions in probing depth (PD), CAL, alveolar bone loss, and inflammatory biomarkers (TNF‐α, IL‐6, CRP). Yilmaz and Görgin [154] found that probiotics combined with a personalized anti‐inflammatory diet yielded the highest improvements in PD (41.5%) and CAL (42.7%), underscoring the synergistic role of nutrition. By contrast, Huo et al. [155] observed that adjunctive *Limosilactobacillus reuteri* did not improve PD or CAL compared with placebo, though it favorably shifted the subgingival microbiota by reducing *Tannerella forsythia* . In a specific population, Jardini et al. [156] showed that *L. reuteri* provided no local benefit in periodontitis patients with type 2 diabetes but improved systemic outcomes by favorably modulating the atherogenic lipoprotein profile, suggesting cardiovascular protective potential. Ramos et al. [157] similarly reported that neither systemic antibiotics (amoxicillin, metronidazole) nor *L. reuteri* provided additional full‐mouth benefits beyond subgingival instrumentation after 90 days, despite improvements in deep pockets and BOP. In contrast, Alkaya et al. [42] demonstrated that daily intake of probiotic ayran containing *L. acidophilus* and *B. bifidum* reduced plaque index, gingival index, BOP, and MMP‐8 levels in an experimental gingivitis model, supporting a protective effect against gingival inflammation.

Exploring immunological pathways, Invernici et al. [49] found that *Bifidobacterium animalis* subsp. *lactis* HN019 as an adjunct reduced plaque and gingival bleeding, enhanced beta‐defensin‐3, TLR4, and CD4 expression in gingival tissues, and exhibited antimicrobial activity against periodontopathogens. Extending these findings, Levi et al. [158] showed that *B. lactis* HN019 lozenges used for 8 weeks in patients with generalized gingivitis significantly reduced BOP and pro‐inflammatory cytokines (IL‐1α, IL‐1β, MCP‐1), with more patients achieving gingival health compared with placebo. Taken together, clinical evidence suggests that probiotic efficacy is strain‐, delivery‐, and context‐dependent. While results with *L. reuteri* are variable and often show some periodontal benefit, *B. lactis* HN019 shows more consistent immunomodulatory and clinical effects, particularly in gingivitis and as an adjunct to conventional periodontal therapy.

Translational barriers for probiotics also include regulatory classification ambiguities, whether they should be regulated as drugs, foods, or medical devices, and challenges in standardizing viable bacterial counts, storage conditions, and shelf‐life stability. Furthermore, optimal strain selection, strain‐specific effects, dosing regimens, timing relative to conventional periodontal therapy, and patient‐to‐patient microbiome differences remain unresolved [149, 150, 151, 152]. Delivery vehicles (e.g., lozenges, yogurt, tablets) also vary widely in their ability to maintain bacterial viability, colonization potential, and therapeutic concentrations within periodontal pockets. Advances in microbiome sequencing, systems biology, and synthetic biology may enable the development of next‐generation probiotics or genetically engineered bacterial consortia with enhanced ecological capability, targeted antimicrobial activity, and immunomodulatory properties tailored to individual patients.

### 4.4 Small Molecules and Targeted Host‐Modulatory Agents

Beyond natural products, host modulators and probiotics, several synthetic small molecules targeting inflammatory and osteoimmune pathways have shown efficacy in preclinical periodontitis models. For a comprehensive overview of the effects of various small‐molecule inhibitors and kinase inhibitors on experimental periodontitis, please refer to the study by de Molon et al. [10]. Examples include chemically modified tetracyclines (e.g., doxycycline derivatives) with matrix metalloproteinase‐inhibitory activity; p38 MAPK inhibitors suppressing proinflammatory cytokine production; and NF‐κB, PI3K/Akt, or JAK/STAT pathway inhibitors attenuating osteoclastogenesis and tissue destruction [10].

Other small molecules, such as complement inhibitors (e.g., AMY‐101 targeting C3), are undergoing early‐phase clinical testing [41, 159]. The recent phase IIa trial of AMY‐101 demonstrated significant reductions in gingival inflammation without major adverse events, underscoring the feasibility of locally delivered complement inhibitors as precision therapeutics. However, their incorporation into local drug delivery systems platforms remains limited, with most early‐phase trials relying on systemic or repeated local injections rather than sustained‐release carriers. Advances in nanotechnology, thermosensitive hydrogels, and biodegradable polymer scaffolds may enable more precise delivery and prolonged pharmacologic action at disease sites while minimizing systemic exposure (refer to Section 5).

Beyond small molecules and biologics, fully synthetic macromolecular inhibitors represent another promising avenue for host modulation. Dendritic polyglycerol sulphates (dPGS) and related co‐polymers (e.g., dendritic poly (glycerol‐caprolactone) sulphate, dPGS‐PCL) are designed to operate via a multivalent binding mechanism, mimicking naturally occurring ligands [160]. These highly sulfated polymers act as potent inhibitors of L‐ and P‐selectin, dampening leukocyte extravasation, and also bind directly to complement factors C3 and C5, thereby inhibiting the generation of pro‐inflammatory anaphylatoxins like C5a. This dual mechanism of action, targeting both cellular adhesion and the complement system, positions dPGS as an innovative, fully synthetic therapeutic class that could be explored in the context of periodontal host modulation, potentially overcoming some of the limitations of biological agents [160].

### 4.5 Device‐Based Adjuncts: Photodynamic Therapy and Lasers

Physical adjuncts such as antimicrobial photodynamic therapy (aPDT) and various laser systems (e.g., diode, Er:YAG, Nd:YAG) have been proposed to enhance bacterial eradication and modulate host inflammatory responses. In vitro and preclinical studies demonstrate that aPDT, combining a photosensitizer with specific light wavelengths to generate cytotoxic reactive oxygen species, reduces periodontal pathogen loads and inflammatory markers [60, 161, 162, 163]. Some clinical trials report additional probing depth reduction and clinical attachment gain when aPDT is used alongside subgingival instrumentation [164, 165], while others find minimal improvements in inflammation [166] or no benefits [167, 168, 169].

Systematic reviews consistently conclude that heterogeneity in photosensitizers, light sources, treatment protocols, and study designs precludes definitive recommendations [170, 171]. Similarly, laser monotherapy shows inconsistent advantages over conventional therapy, with most guidelines refraining from endorsing routine clinical use. Importantly, both aPDT and laser applications face translational barriers related to equipment costs, operator training, standardization of energy parameters, and demonstration of long‐term clinical superiority over mechanical debridement alone.

The adjunctive strategies outlined above illustrate both the progress and persistent challenges in translating biological innovation into clinical outcomes. Advances in translational modeling directly shape the evolution of next‐generation adjunctive therapies for periodontitis. Refinements in animal and ex vivo models, together with improved understanding of host–microbiome interactions, enable more predictive testing of pharmacologic, biologic, and device‐based interventions. These methodological innovations provide the experimental precision required to design delivery systems, drug formulations, and regenerative platforms that better mimic human pathophysiology. Consequently, the transition from mechanistic discovery to clinical translation is becoming increasingly seamless, supporting the development of adjunctive therapies that are not only biologically rational but also clinically viable. To move beyond isolated therapeutic advances, future research must integrate these approaches into cohesive, patient‐centered frameworks supported by new technologies and data‐driven decision tools. The next sections therefore focus on emerging translational strategies, particularly bioresponsive delivery platforms and artificial intelligence, that may redefine how adjunctive therapies are selected and applied in clinical periodontology.

## 5 Adjunctive Therapeutics Innovations

Unlike other chronic diseases characterized by steady progression, periodontitis tends to occur in episodic bursts of activity, interspersed with long periods of quiescence [172]. While gingival inflammation is histologically characterized by loss of collagen and destruction of extracellular matrix to accommodate the inflammatory infiltrate, bone resorption in periodontitis is not constant—it is stochastic and localized, driven by acute inflammatory exacerbations followed by tissue remodeling phases [10]. The episodic pattern of bone destruction in periodontitis raises important questions about whether there may be a critical window of opportunity for therapeutic intervention. Although periodontitis is typically diagnosed at the patient level, classified simply as present or absent rather than by ongoing disease activity, this approach makes it difficult to determine the optimal time for treatment. Nevertheless, the timing of adjunctive therapies aimed at modulating inflammation, preventing bone loss, or controlling microbial factors may be pivotal for achieving the best outcomes, potentially depending on the cyclical nature of tissue destruction [10].

One of the main challenges in testing new adjunctive therapies in animal models is ensuring that the delivery method can be translated to humans. For example, drugs intended for oral administration in humans are often delivered to animals via oral gavage, which may alter absorption. Similarly, because animals cannot gargle or rinse, alternative methods are needed to test mouthwashes. For instance, (i) topical application with microbrushes or cotton swabs, allowing direct delivery of the formulation to the gingival or tooth surfaces; (ii) local irrigation or dripping systems, where small volumes of the test solution are applied into the oral cavity or periodontal pockets using microsyringes; (iii) gel or mucoadhesive formulations, derived from mouthwash components but optimized for adhesion to mucosal or dental surfaces, prolonging contact time in the oral cavity; and (iv) incorporation into drinking water, when appropriate for systemic evaluation, though this method provides less control over dosage and exposure duration. Additionally, some adjustments related to animal size must be performed when incorporating a potential human treatment—this includes adjusting dose, time or instruments, such as laser or light tips, for example. All these modifications affect the direct clinical application of preclinical evidence, as new standardizations should be further performed in humans.

### 5.1 Advances in Local Drug Delivery Systems for Periodontal Therapy

**FIGURE 4:**
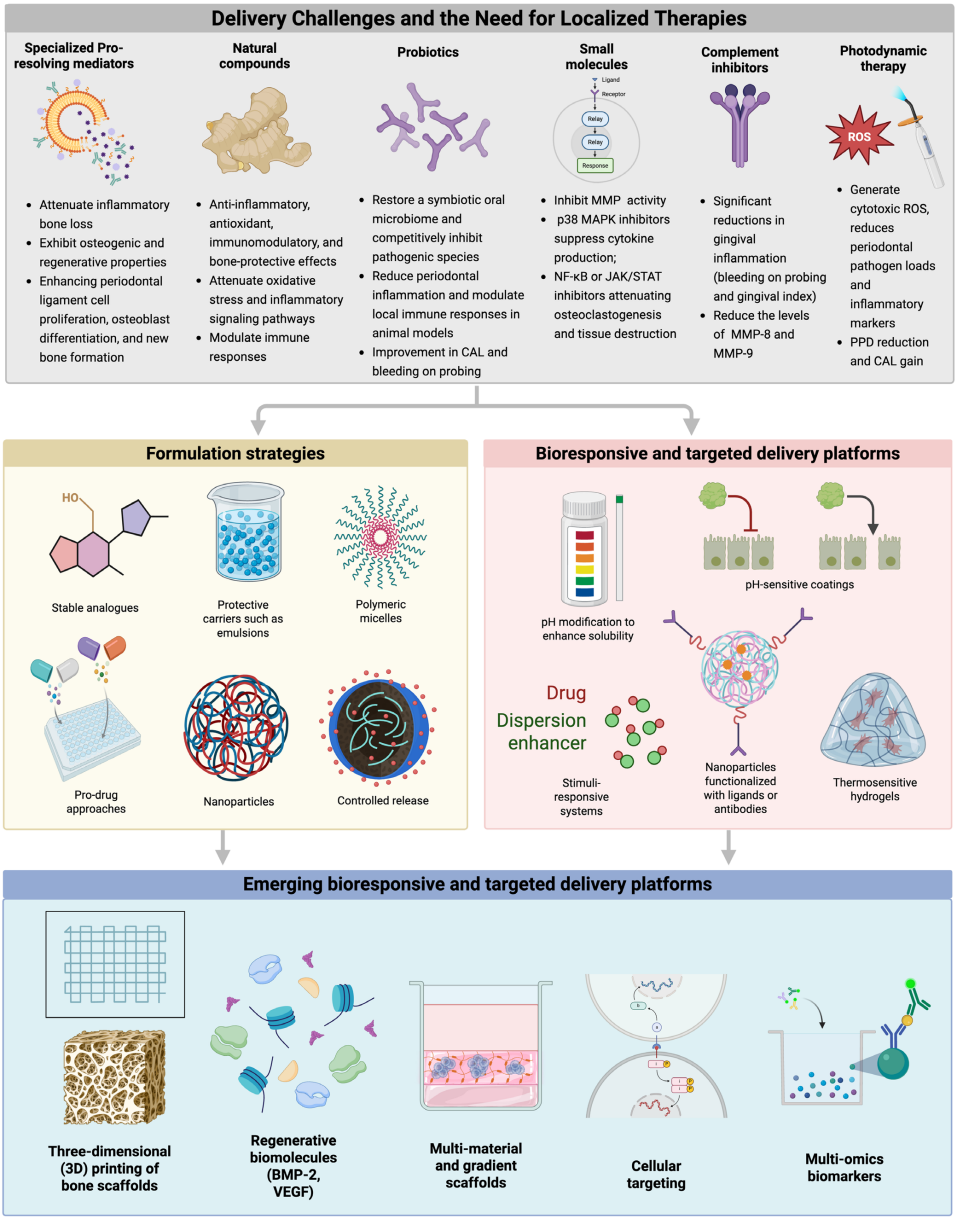
Delivery challenges and emerging strategies for localized therapies in periodontitis. The schematic summarizes the therapeutic approaches and delivery platforms aiming to overcome challenges associated with localized treatment of periodontitis. Top panel: Current adjunctive therapies include SPMs, natural compounds (e.g., curcumin, resveratrol), probiotics, small molecules, complement inhibitors, and photodynamic therapy, each targeting inflammation, bone loss, microbial dysbiosis, or tissue regeneration. Middle panels: Formulation strategies involve stable analogs, protective carriers (e.g., emulsions, polymeric micelles), prodrug approaches, nanoparticles, and controlled release systems to enhance drug stability and sustained local delivery. Bioresponsive and targeted delivery platforms incorporate pH‐sensitive systems, thermoresponsive hydrogels, nanoparticle surface modifications (e.g., antibody conjugation), and dispersion enhancers to achieve site‐specific and stimulus‐responsive release. Bottom panel: Emerging approaches include three‐dimensional (3D) printing of scaffolds, regenerative biomolecules (e.g., BMP‐2, VEGF), multi‐material gradient scaffolds, cellular targeting strategies, and multi‐omics biomarkers, representing next‐generation bioresponsive platforms for precision periodontal therapy.

Local drug delivery systems (LDDS) have been developed to deliver therapeutic agents directly to the periodontal pocket, where they can act in close proximity to the sites of inflammation and tissue destruction. Various LDDS platforms have been explored, including mouthwashes, antimicrobial gels, fibers impregnated with antibiotics, biodegradable microspheres, thermosensitive or mucoadhesive hydrogels, and nanoscale carriers designed to release their payload in a controlled manner [55, 81, 82, 173, 174]. These systems are intended to achieve high local drug concentrations while minimizing systemic exposure and adverse effects. By prolonging drug residence within the pocket (when necessary, at all) and reducing the need for frequent systemic dosing, LDDS theoretically improve efficacy while enhancing patient safety. Furthermore, advanced polymeric nanocarriers have been recently investigated to overcome some limitations of delivery methods. Formulation strategies, and bioresponsive and targeted delivery platforms for LDDS are discussed in the following subsections (Figure 4).

### 5.2 Bioinspired Adhesive Systems

Recent innovations in polymer science have yielded nanocarriers with combined functionalities, such as intrinsic anti‐inflammatory activity and robust mucoadhesion, which are highly desirable for periodontal applications. Dendritic poly (glycerol‐caprolactone) sulphate (dPGS‐PCL) represents one such platform. This biodegradable polymer is designed with cleavable ester bonds in its backbone for enzymatic degradation, addressing bioaccumulation concerns associated with earlier non‐degradable variants [175]. In ex vivo and in vitro models, dPGS‐PCL demonstrated rapid penetration into porcine masticatory mucosa within seconds and exhibited a potent, specific downregulatory effect on the pro‐inflammatory chemokine IL‐8 (CXCL8) in gingival epithelial cells and 3D mucosal organoids [175]. Given IL‐8's central role in neutrophil recruitment, this intrinsic anti‐inflammatory action, coupled with its drug‐carrying capacity, positions dPGS‐PCL as a promising dual‐action therapeutic system for periodontitis.

Beyond lipid‐based mediators like SPMs, innovative synthetic nanocarrier platforms are also being developed to overcome the challenges of topical oral drug delivery. The core‐multishell (CMS) nanocarrier is one such dendritic polymer‐based system, consisting of a hyperbranched polyglycerol core, a lipophilic inner shell, and a hydrophilic poly(ethylene glycol) outer shell [176, 177]. This architecture allows for the efficient encapsulation of both hydrophilic and hydrophobic drugs. Preclinical studies have demonstrated that ester‐based CMS nanocarriers (e.g., CMS 10‐E‐15‐350) exhibit excellent biocompatibility, rapidly penetrate oral mucosal tissues within minutes, and do not compromise the epithelial barrier or induce a pro‐inflammatory response [177]. Crucially, when loaded with dexamethasone, the CMS nanocarrier provides superior anti‐inflammatory efficacy in reducing IL‐6 and IL‐8 levels in human 3D gingival mucosal models compared to a conventional dexamethasone cream [178, 179]. A significant recent advancement involves functionalizing the CMS nanocarrier with catechol groups, inspired by mussel adhesion proteins. This modification dramatically enhances mucoadhesion even in the presence of saliva, a major hurdle for oral topical therapies, and leads to improved drug delivery and anti‐inflammatory effects under dynamic, clinically relevant conditions [179]. The CMS platform thus represents a highly tunable and promising LDDS for the targeted delivery of host‐modulatory agents in periodontitis.

### 5.3 Bioresponsive and Targeted Delivery Platforms

Emerging bioresponsive and targeted delivery platforms hold promise for clinical translation. Stimuli‐responsive systems can release drugs in response to environmental cues that mirror active disease, such as the acidic microenvironment generated by inflammatory infiltrates or the presence of specific bacterial enzymes [180, 181, 182]. pH‐sensitive coatings, for example, can trigger drug release preferentially in inflamed periodontal pockets, while thermosensitive hydrogels transition from liquid to gel at body temperature to provide prolonged local retention [183]. Nanoparticles functionalized with ligands or antibodies may allow precise delivery of small molecules, peptides, or nucleic acid therapeutics to key cell types, including activated macrophages or osteoclast precursors, potentially enhancing both anti‐inflammatory and bone‐preserving effects [180, 181, 182].

### 5.4 Three‐Dimensional (3D) Bone Printing

Complementing these bioresponsive strategies, three‐dimensional (3D) printing of bone scaffolds has recently emerged as a transformative approach for periodontal regeneration. Not only does it allow for patient‐specific geometries and controlled porosity to promote osteoconduction and vascularization, but it also enables the spatiotemporal incorporation of regenerative biomolecules like BMP‐2 and VEGF. For instance, Freeman et al. developed 3D‐printed hydrogel‐based scaffolds with temporally patterned release of BMP‐2 and VEGF, improving bone formation in critical defects [184]. Another study by Liu et al. utilized 3D‐printed titanium scaffolds loaded with VEGF/BMP‐2 microspheres, achieving both improved osteogenesis and scaffold integration [185]. A recent systematic review underscores the preclinical success of 3D‐printed scaffolds in achieving periodontal regeneration in animal models [186]. Moreover, 3D printing facilitates the incorporation of bioactive molecules, such as growth factors, antibiotics, or even the SPMs discussed earlier, directly into the scaffold matrix for spatiotemporally controlled release. Some platforms integrate stem cells or exosomes within the printed constructs, transforming them into osteogenic and immunomodulatory niches capable of simultaneously addressing bone loss and inflammation.

Recent studies also highlight the feasibility of multi‐material and gradient scaffolds, where the outer layers provide mechanical support while the inner compartments release therapeutics or house regenerative cells [187]. When combined with stimuli‐responsive drug delivery systems, 3D‐printed scaffolds hold the potential to achieve precision‐guided regeneration, delivering biologically active molecules on demand while providing the structural template necessary for periodontal tissue repair.

### 5.5 Practical Limitations of Advanced Delivery Strategies

The clinical translation of these advanced delivery strategies remains in an early phase. Manufacturing complexity, batch‐to‐batch reproducibility, and regulatory requirements for safety and quality pose significant difficulties. Scalable production that maintains the physicochemical properties and bioactivity of these carriers is essential for regulatory approval and clinical adoption. Additionally, long‐term biocompatibility, potential for local irritation or hypersensitivity, and the risk of off‐target effects must be rigorously evaluated. From a clinical perspective, adoption will also require evidence that these technologies provide meaningful improvement in long‐term periodontal outcomes, such as tooth retention, reduced recurrence, or improved oral function, rather than only short‐term surrogate endpoints.

Moreover, retention of locally delivered formulations in the dynamic oral environment remains a major obstacle. Mechanical forces from mastication, routine oral hygiene practices, constant salivary flow, and the natural clearance of gingival crevicular fluid all contribute to premature drug loss. Even advanced mucoadhesive formulations or in situ gelling systems often provide limited residence times, compromising sustained therapeutic effects. Patient‐related variables, including smoking, inconsistent oral hygiene, or suboptimal adherence to post‐application instructions, further reduce real‐world efficacy.

Additionally, many currently available LDDS formulations were originally developed as antimicrobial agents, primarily designed to suppress bacterial load rather than directly modulate host immune responses, promote tissue regeneration, or rebalance the microbiome. Translational progress requires delivery systems capable of reliably carrying a broad spectrum of adjunctive therapeutics, including anti‐inflammatory small molecules, host‐modulatory biologics, probiotics, and natural compounds. However, issues related to drug stability, controlled release, and preservation of bioactivity under oral conditions have hindered clinical adoption [173, 181, 188, 189, 190].

Overall, the promise of LDDS lies in integrating multiple therapeutic classes, SPMs, natural products, probiotics, small molecules, and future biologics, into stable, biocompatible, and patient‐friendly delivery systems. Achieving this goal will require optimization of formulation chemistry, release kinetics, and mechanical retention, alongside well‐powered clinical trials to define therapeutic windows, dosing schedules, and long‐term safety profiles. Such multidimensional approaches have the potential to transform LDDS from experimental tools into clinically viable adjunctive therapies for periodontitis. Table 3 summarizes the translational readiness of adjunctive therapies for periodontitis.

**TABLE 3:** Comparative translational readiness of adjunctive therapies for periodontitis.

Therapy	Preclinical promise	Current clinical evidence	Main barrier (bioavailability, cost, trial design, regulatory)
Specialized Pro‐resolving Mediators	Strong anti‐inflammatory and pro‐regenerative effects in animal models; well‐characterized pathways	Early‐phase pilot human studies show safety; limited efficacy data	Short half‐life, delivery challenges, regulatory classification as biologics
Probiotics	Demonstrated modulation of biofilm and inflammation in vitro and animal models	Multiple small RCTs show reduction in probing depth and bleeding	Strain specificity, heterogeneity of formulations, trial reproducibility
Natural Products (e.g., curcumin, resveratrol, tanshinones)	Potent antioxidant and anti‐inflammatory effects in preclinical models	Scattered small human studies; inconsistent clinical benefits	Poor bioavailability, lack of standardized dosing, regulatory hurdles
Photodynamic Therapy	Effective microbial killing, biofilm disruption, and host modulation preclinically	Several RCTs with modest but consistent adjunctive improvements	Device cost, standardization of protocols, operator variability
Hydrogels (injectable/bioresponsive)	Excellent controlled release and regenerative support in preclinical models	Early clinical feasibility studies ongoing	Manufacturing complexity, long‐term safety, regulatory approval
Nanocarriers (liposomes, nanoparticles)	Enhanced drug stability and targeting in vitro and animal studies	Very limited clinical data; mostly preclinical	Scale‐up challenges, safety/toxicity concerns, regulatory complexity
3D‐Printed Scaffolds	Strong potential for guided regeneration and personalized therapy in animals	No established periodontal RCTs; mostly experimental	Cost, reproducibility, regulatory approval for clinical translation

In summary, while local delivery offers an attractive strategy to reconcile efficacy with safety in adjunctive periodontal therapy, it is constrained by biological, mechanical, and translational challenges. The next generation of LDDS will likely combine bioresponsive release, precise cellular targeting, and enhanced retention to create therapies that can be integrated seamlessly into clinical practice. Success in this domain will depend on multidisciplinary efforts that span materials science, pharmacology, microbiology, and clinical periodontology, ultimately aiming to transform promising preclinical technologies into reliable, patient‐centered therapeutic options. Integration of multi‐omics biomarkers, advanced drug delivery systems, and precision medicine approaches leveraging artificial intelligence may enable personalized selection of adjunctive therapies based on individual microbiologic, inflammatory, and genetic signatures. Furthermore, combination strategies, such as pairing probiotics with host‐modulatory agents or incorporating natural compounds into bioresponsive delivery systems, hold promise for synergistic effects on microbial dysbiosis, inflammation resolution, and periodontal regeneration. Understanding these delivery constraints provides the foundation for evaluating the translational readiness of emerging adjunctive interventions.

The adjunctive strategies outlined above illustrate both the progress and persistent challenges in translating biological innovation into clinical outcomes. To move beyond isolated therapeutic advances, future research must integrate these approaches into cohesive, patient‐centered frameworks supported by new technologies and data‐driven decision tools. The next section therefore focuses on emerging translational strategies, particularly bioresponsive delivery platforms and artificial intelligence, that may redefine how adjunctive therapies are selected and applied in clinical periodontology.

## 6 Emerging Translational Strategies and Clinical Integration

**FIGURE 5:**
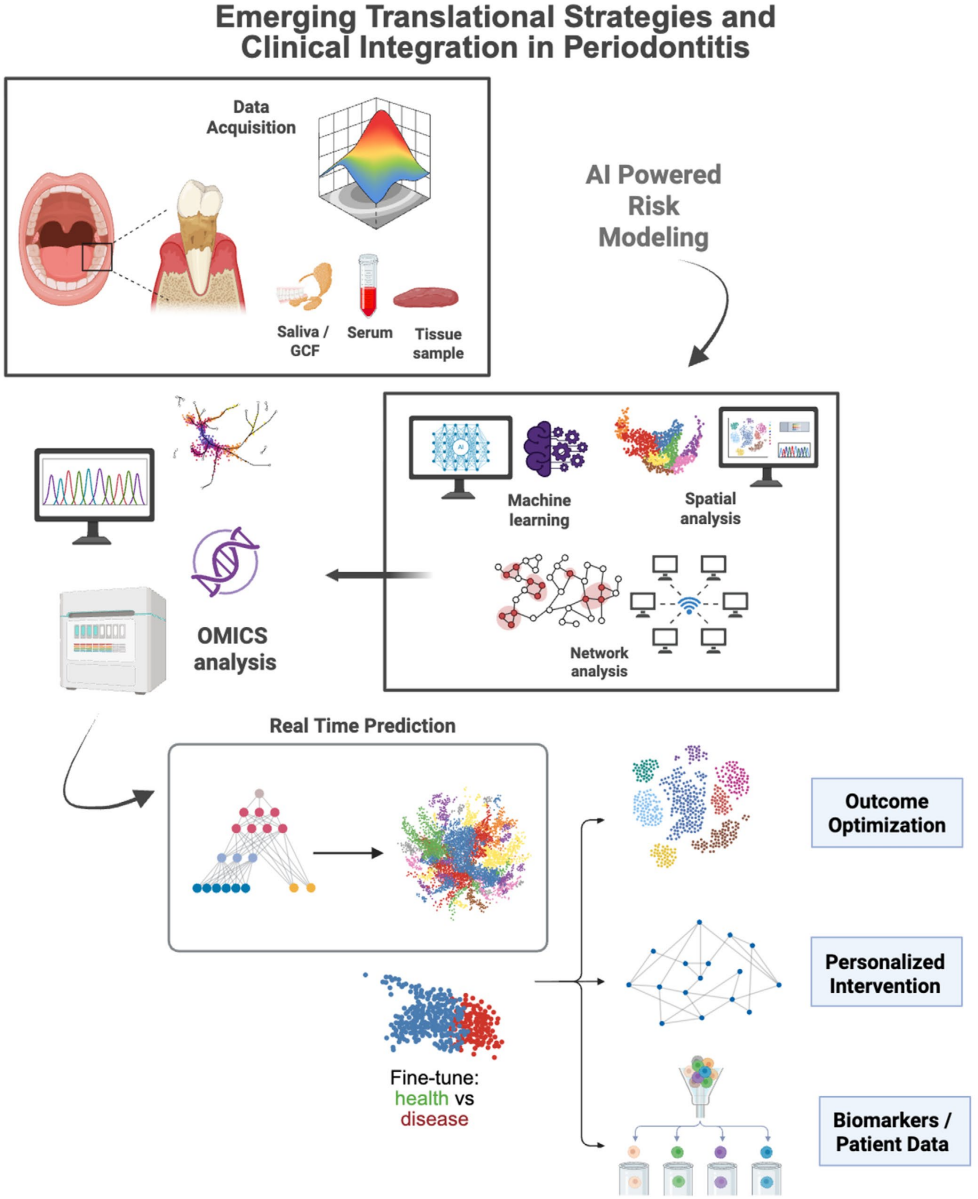
Artificial intelligence (AI)‐driven integration of precision adjunctive therapies in periodontitis management. Data acquisition from clinical, imaging, and biomarker sources feeds into AI‐powered risk modeling to predict disease activity and progression in real time. Machine learning algorithms fine‐tune predictions to discriminate healthy versus diseased states, enabling the development of personalized interventions. Integration of patient‐specific data, including biomarkers and risk profiles, supports outcome optimization by tailoring adjunctive therapies to individual needs, advancing precision periodontics through data‐informed decision‐making.

Beyond biological and logistical hurdles, improving clinical translation also involves reducing animal research in an evolving ethically driven society. For that purpose, alternative models that provide smarter disease management strategies, such as in vitro tissue models, bioinformatics prediction models and artificial intelligence (AI), come into play. The clinical relevance of adjunctive therapies in periodontitis is being redefined by the rapid integration of AI into diagnostics, risk prediction, and personalized care (Figure 5) [191, 192, 193, 194, 195, 196]. One of the greatest challenges in periodontology remains the precise characterization of disease activity and timely identification of patients who will benefit most from adjunctive interventions. Traditional tools, clinical probing depths, bleeding on probing, and radiographic imaging provide static, retrospective assessments of cumulative tissue loss rather than real‐time indicators of active disease [197]. AI‐enabled platforms, combining multi‐omic data with longitudinal clinical records, now have the potential to detect subclinical inflammation, predict imminent flare‐ups, and recommend targeted intervention windows before irreversible tissue destruction occurs [198, 199]. In sum, AI might help researchers to better identify when and for whom to use adjunctive therapies.

Emerging machine learning algorithms trained on biomarkers from gingival crevicular fluid, saliva, and blood, as well as host and microbial profiles, can identify complex disease signatures that are invisible to conventional diagnostics [198, 199] (Figure 5). These AI‐driven risk models integrate genetic polymorphisms, immune phenotypes, epigenetic modifications, and environmental exposures to stratify patients according to their likelihood of rapid disease progression or poor response to standard therapy. By continuously updating risk profiles as new data are acquired, AI facilitates dynamic disease monitoring and supports the concept of precision periodontics, where adjunctive therapies are tailored to individual trajectories rather than administered uniformly [191, 192, 193, 194, 195].

The interaction between systemic conditions, such as diabetes mellitus, obesity, and smoking, and periodontal inflammation adds further complexity to clinical decision‐making. AI systems can model multidimensional data streams linking systemic inflammation, metabolic dysregulation, and local periodontal pathology, enabling the identification of therapeutic synergies where adjunctive treatments simultaneously improve oral health and systemic outcomes. For instance, predictive models may pinpoint diabetic patients who would derive dual benefit from anti‐inflammatory adjuncts by mitigating both periodontal breakdown and systemic cytokine spillover, potentially enhancing glycemic control [191, 192, 193, 194, 195].

Risk stratification is evolving from static staging and grading toward real‐time, AI‐assisted risk forecasting. Reinforcement learning approaches can adapt “treat‐to‐target” paradigms from other chronic diseases to periodontology, using continuously updated data to determine when to escalate or de‐escalate adjunctive interventions. AI‐derived endpoints, incorporating patient‐centered outcomes like long‐term tooth retention, masticatory function, and quality of life, can provide clinically meaningful metrics that extend beyond traditional measures such as probing depth reduction, enabling more accurate cost‐effectiveness analyses and regulatory approval pathways [200].

The timing of adjunctive therapy is another critical aspect of its clinical utility. There is ongoing debate whether to deploy adjuncts during overtly active disease in a targeted fashion at sites showing molecular or clinical signs of activity or prophylactically in high‐risk individuals to prevent exacerbations. Without real‐time indicators of activity, prophylactic use risks overtreatment and unnecessary exposure, while delayed application after established destruction limits regenerative potential. Point‐of‐care platforms and multi‐omic profiling, combined with machine learning algorithms, are being developed to predict flare‐ups and define windows of therapeutic opportunity, potentially enabling intervention before irreversible tissue loss occurs [201]. AI‐powered point‐of‐care platforms, integrating molecular biosensors with predictive analytics, can forecast flare‐ups and define optimal therapeutic windows. Such systems promise to transform adjunctive therapy from a reactive to a proactive, precision‐guided intervention [191, 192, 193, 194, 195].

Personalizing adjunctive therapy also requires identifying responders versus non‐responders. Variability in host response, microbial composition, and pharmacogenomics means that some patients derive meaningful benefit while others do not, and indiscriminate application of adjuncts can dilute perceived effectiveness in population‐level studies. Precision diagnostics can help select patients likely to respond to specific modalities, whether host modulators, local antimicrobials, or regenerative biologics, thereby optimizing resource allocation and minimizing unnecessary side effects. In parallel, stewardship principles, particularly for systemic antimicrobials, demand that adjunctive use be justified by stratified risk profiles and supported by evidence of likely benefit [111]. This aligns with antimicrobial stewardship principles, where systemic adjuncts are prescribed selectively based on AI‐derived evidence of likely efficacy rather than population‐level averages.

Economic and implementation considerations further underscore clinical relevance. Adjunctive therapies, especially biologics and advanced delivery systems, can be costly, and their added value must be weighed against standard mechanical therapy in terms of long‐term outcomes, prevention of tooth loss, reduction in downstream rehabilitative needs, and mitigation of systemic comorbidities. Health economic evaluations that incorporate direct costs, indirect savings from preserved oral function, and quality‐adjusted life years will be essential to justify their integration into routine care, particularly in resource‐constrained settings. Equitable access, clinician education on interpretation of novel diagnostics, and standardization of biomarker assays are practical barriers that must be addressed to translate precision adjunctive approaches broadly [111]. AI‐enabled health economic models can simulate long‐term outcomes, quantifying not only direct costs but also indirect savings through preserved oral function, reduced rehabilitative needs, and mitigation of systemic comorbidities. By incorporating quality‐adjusted life years (QALYs) and real‐world cost data, these models can support policy‐level decisions on reimbursement and equitable access, especially in resource‐limited settings.

Finally, embedding adjunctive therapies into AI‐driven, longitudinal care ecosystems allows for continuous adaptation of maintenance intervals, re‐evaluation thresholds, and escalation protocols based on evolving risk profiles. Integrating periodontal care with primary and specialty care through interoperable AI platforms facilitates a holistic oral‐systemic health strategy, ensuring that adjunctive interventions contribute not only to local periodontal stability but also to broader preventive health outcomes.

Collectively, these emerging tools signal a paradigm shift toward precision periodontics, where interventions are guided by molecular insights and predictive analytics. Yet, realizing this vision will require coordinated efforts in research design, delivery optimization, and policy development. The final section outlines key research priorities and translational pathways needed to transform these advances into clinically viable, accessible solutions.

## 7 Future Directions and Research Priorities in Translational Research

While methodological and ethical limitations in animal research remain significant, future efforts must focus on integrating alternative models and technologies that better capture the complexity of human periodontitis. By incorporating these next‐generation tools (as described below), translational research can become both ethically responsible and scientifically robust, ensuring that preclinical discoveries are validated in systems that more closely replicate human pathophysiology. To advance the clinical utility of adjunctive therapies for periodontitis in translational research, several areas warrant attention:

### 7.1 Development of Better and Alternative Models

While improved animal models that closely mimic human periodontitis remain valuable, there is a growing emphasis on reducing and ultimately phasing out animal experimentation whenever feasible. Novel technologies such as organ‐on‐a‐chip platforms, 3D tissue‐engineered gingival constructs, and microfluidic “lab‐on‐a‐chip” systems offer promising alternatives by enabling high‐throughput, mechanistic, and patient‐specific studies under controlled conditions [202, 203, 204]. These platforms can reproduce host–microbe interactions, inflammatory cascades, and drug responses with increasing fidelity while addressing ethical concerns. Although they cannot yet fully replicate the systemic complexity and chronicity of human disease, integrating these next‐generation in vitro models into translational pipelines should be a priority for the field. Within this evolving landscape, the 3Rs principle, Replacement, Reduction, and Refinement, provides an essential ethical framework for guiding preclinical research [205, 206, 207]. Replacement advocates for the use of alternative in vitro systems, organoids, microfluidic devices, or computational simulations whenever they can yield comparable mechanistic insights; Reduction emphasizes minimizing animal use through robust experimental design, statistical rigor, and data sharing; and Refinement seeks to improve welfare through optimized housing, anesthesia, analgesia, and humane endpoints. Together, these strategies balance scientific advancement with ethical responsibility, enhancing reproducibility, translational relevance, and societal trust in preclinical research.

### 7.2 Biomarker Discovery and Validation

Identifying reliable biomarkers that accurately reflect the onset, activity, and progression of periodontitis is critical for enabling real‐time monitoring and precision‐targeted interventions. Current diagnostic tools primarily detect historical tissue destruction rather than ongoing disease activity, underscoring the urgent need for dynamic molecular indicators. To this end, multi‐omics approaches, including proteomics, transcriptomics, metabolomics, and increasingly, microbiomics, should be systematically applied in longitudinal human cohorts to capture the temporal fluctuations in host–microbe interactions and inflammatory mediators associated with disease exacerbation and remission. The ultimate goal is to translate these findings into validated biomarker panels for point‐of‐care diagnostics, enabling clinicians to assess inflammatory burden, risk of disease progression, and treatment responsiveness chairside. Integration with biosensor technologies, salivary diagnostics, and even wearable platforms could further revolutionize disease monitoring, paving the way for personalized, minimally invasive periodontal care.

### 7.3 Optimization of Delivery Systems

Future research should prioritize the design and optimization of advanced LDDS that provide precise, sustained, and targeted therapeutic action within periodontal tissues. Next‐generation platforms aim to address limitations related to drug stability, release kinetics, and patient compliance. Bioresponsive formulations, capable of releasing drugs in response to disease‐associated cues such as pH changes, reactive oxygen species, or bacterial enzymes, represent a promising direction. By aligning drug release with local inflammatory or infectious triggers, these systems can achieve on‐demand therapy, reducing drug waste and minimizing off‐target effects. Controlled‐release technologies utilizing polymeric matrices, liposomes, or thermosensitive hydrogels further enhance local retention time, enabling prolonged therapeutic exposure without repeated clinical applications. Emerging technologies such as 3D‐printed drug‐eluting scaffolds allow for patient‐specific geometries that fit complex periodontal defects, combining regenerative potential with controlled drug delivery. Similarly, nanocarrier systems, including polymeric nanoparticles, dendrimers, and lipid‐based nanovesicles, offer the possibility of cell‐specific targeting, for example, directing anti‐inflammatory or pro‐regenerative agents toward activated macrophages, osteoclast precursors, or stem/progenitor cells involved in tissue repair.

Importantly, successful translation will require delivery platforms to be clinically feasible, cost‐effective, and minimally invasive, with manufacturing processes compatible with regulatory requirements and scalable production. Ultimately, integrating theranostic capabilities, where delivery systems also provide real‐time monitoring of drug release or disease activity, could further transform personalized periodontal therapy.

### 7.4 Cost–Benefit Analyses

Given the considerable financial burden associated with biologics and emerging small‐molecule therapies, rigorous health economic assessments are essential to determine their feasibility in routine periodontal care. These analyses should go beyond simple drug pricing and incorporate broader perspectives on cost‐effectiveness, including long‐term outcomes for both oral and systemic health. For instance, preventing tooth loss and the subsequent need for prosthetic rehabilitation could offset initial expenditures, while mitigating systemic inflammation may reduce the risk and healthcare costs associated with comorbid conditions such as diabetes and cardiovascular disease. Additionally, cost–benefit frameworks should evaluate quality of life improvements, productivity gains from reduced disability, and the potential reduction in healthcare utilization at the population level. Only by demonstrating favorable cost‐effectiveness ratios can these novel therapies be realistically integrated into public health strategies and clinical guidelines.

### 7.5 Interdisciplinary Collaboration

Successfully bridging the translational gap between preclinical innovation and clinical implementation will require close collaboration across diverse scientific and clinical disciplines. Periodontologists provide insights into disease pathogenesis and patient‐centered outcomes, while pharmacologists contribute expertise on drug delivery, pharmacokinetics, and safety. Materials scientists and bioengineers can design innovative drug carriers, scaffolds, and biomaterials that enhance local delivery and therapeutic efficacy. Health economists are crucial in assessing the economic sustainability of new approaches, and computational modelers can predict biological responses, optimize dosing regimens, and refine trial designs. Establishing multidisciplinary research consortia, supported by international collaborations and funding agencies, can create a synergistic environment where basic discoveries are rapidly translated into clinically viable adjunctive therapies. Such integrated efforts not only accelerate innovation but also ensure that novel strategies are both biologically effective and practically applicable in real‐world healthcare settings.

## 8 Conclusion

The management of periodontitis is entering a new era where adjunctive therapies can extend the benefits of mechanical debridement by modulating host responses, controlling inflammation, and promoting regeneration. Yet, translation remains challenging due to episodic disease activity, limited predictive models, pharmacokinetic constraints, and economic feasibility.

Short‐term priorities should focus on standardizing animal model protocols, harmonizing outcome measures, and incorporating comorbidities into preclinical systems. Medium‐term goals include validating biomarkers of activity, testing bioresponsive delivery platforms, and conducting trials with patient‐centered endpoints. Long‐term directions should integrate adjunctive therapies into precision medicine frameworks supported by artificial intelligence and health‐economic validation, ensuring sustainable adoption into public health strategies. By aligning innovation with clinical feasibility, the field can move closer to delivering adjunctive therapies that improve not only clinical parameters but also the lives of individuals with periodontitis.

## Author Contributions

Conceptualization: R.S.M., W.T. and T.E.V.D. Methodology: R.S.M., J.P.S., E.D.A., W.T. and T.E.V.D. Validation: R.S.M., J.P.S., E.D.A., W.T. and T.E.V.D. Investigation: R.S.M., J.P.S., E.D.A., W.T. and T.E.V.D.; Writing – original draft preparation: R.S.M., J.P.S., E.D.A., W.T. and T.E.V.D. Writing – review and editing: R.S.M., J.P.S., E.D.A., W.T., and T.E.V.D. Funding acquisition: R.S.M. Supervision: R.S.M. All authors have read and agreed to the published version of the manuscript.

## Funding

Rafael Scaf de Molon is currently supported by a grant provided by the Sao Paulo Research Foundation—FAPESP. Grant #2023/15750‐7.

## Conflicts of Interest

The authors declare no conflicts of interest.

## Data Availability

Data sharing not applicable to this article as no datasets were generated or analysed during the current study.

## References

[1] S. D. Dos Anjos , T. C. C. de Meiros , R. M. Ferro , et al., “Occurrence of Periodontal Diseases According to the ACES 2018 Classification Framework and the CDC/AAP Definition: A Cross‐Sectional Study in a Major Brazilian City,” Journal of Clinical Periodontology 51, no. 9 (2024): 1178–1187. 39128863 10.1111/jcpe.14035

[2] D. F. Kinane , P. G. Stathopoulou , and P. N. Papapanou , “Periodontal Diseases,” Nature Reviews Disease Primers 3 (2017): 17038. 10.1038/nrdp.2017.38 28805207

[3] M. S. Tonetti , S. Jepsen , L. Jin , and J. Otomo‐Corgel , “Impact of the Global Burden of Periodontal Diseases on Health, Nutrition and Wellbeing of Mankind: A Call for Global Action,” Journal of Clinical Periodontology 44, no. 5 (2017): 456–462. 28419559 10.1111/jcpe.12732

[4] M. X. Chen , Y. J. Zhong , Q. Q. Dong , H. M. Wong , and Y. F. Wen , “Global, Regional, and National Burden of Severe Periodontitis, 1990‐2019: An Analysis of the Global Burden of Disease Study 2019,” Journal of Clinical Periodontology 48, no. 9 (2021): 1165–1188. 34101223 10.1111/jcpe.13506

[5] N. J. Kassebaum , E. Bernabe , M. Dahiya , B. Bhandari , C. J. Murray , and W. Marcenes , “Global Burden of Severe Periodontitis in 1990‐2010: A Systematic Review and Meta‐Regression,” Journal of Dental Research 93, no. 11 (2014): 1045–1053. 25261053 10.1177/0022034514552491 PMC4293771

[6] G. G. Nascimento , S. Alves‐Costa , and M. Romandini , “Burden of Severe Periodontitis and Edentulism in 2021, With Projections up to 2050: The Global Burden of Disease 2021 Study,” Journal of Periodontal Research 59, no. 5 (2024): 823–867. 39192495 10.1111/jre.13337

[7] R. S. de Molon , E. D. de Avila , J. A. Cirelli , and J. P. Steffens , “Periodontal Research Contributions to Basic Sciences: From Cell Communication and Host‐Parasite Interactions to Inflammation and Bone Biology,” Biocell 46, no. 3 (2022): 633–638.

[8] G. Hajishengallis , “Immunomicrobial Pathogenesis of Periodontitis: Keystones, Pathobionts, and Host Response,” Trends in Immunology 35, no. 1 (2014): 3–11. 24269668 10.1016/j.it.2013.09.001 PMC3947349

[9] T. E. van Dyke , G. Baima , and M. Romandini , “Periodontitis: Microbial Dysbiosis, Non‐Resolving Inflammation, or Both?,” Journal of Periodontal Research (2025), 10.1111/jre.13424. 40657987

[10] R. S. de Molon , R. Vernal , G. E. Oliveira , et al., “Inflammatory Bone Loss and Signaling Pathways in Periodontitis: Mechanistic Insights and Emerging Therapeutic Strategies,” Bone Research (2025), 10.1038/s41413-025-00478-1. PMC12764867 41484074

[11] G. Baima , M. Arce , M. Romandini , and T. van Dyke , “Inflammatory and Immunological Basis of Periodontal Diseases,” Journal of Periodontal Research (2025), 10.1111/jre.70040. 41065279

[12] G. V. O. Fernandes , G. A. Mosley , W. Ross , A. Dagher , B. Martins , and J. C. H. Fernandes , “Revisiting Socransky's Complexes: A Review Suggesting Updated New Bacterial Clusters (GF‐MoR Complexes) for Periodontal and Peri‐Implant Diseases and Conditions,” Microorganisms 12, no. 11 (2024): 2214. 39597602 10.3390/microorganisms12112214 PMC11596145

[13] G. Hajishengallis , “Periodontitis: From Microbial Immune Subversion to Systemic Inflammation,” Nature Reviews. Immunology 15, no. 1 (2015): 30–44. 10.1038/nri3785 PMC4276050 25534621

[14] R. J. Lamont and G. Hajishengallis , “Polymicrobial Synergy and Dysbiosis in Inflammatory Disease,” Trends in Molecular Medicine 21, no. 3 (2015): 172–183. 25498392 10.1016/j.molmed.2014.11.004 PMC4352384

[15] S. S. Socransky , A. D. Haffajee , M. A. Cugini , C. Smith , and R. L. Kent, Jr. , “Microbial Complexes in Subgingival Plaque,” Journal of Clinical Periodontology 25, no. 2 (1998): 134–144. 9495612 10.1111/j.1600-051x.1998.tb02419.x

[16] L. Abusleme , A. K. Dupuy , N. Dutzan , et al., “The Subgingival Microbiome in Health and Periodontitis and Its Relationship With Community Biomass and Inflammation,” ISME Journal 7, no. 5 (2013): 1016–1025. 23303375 10.1038/ismej.2012.174 PMC3635234

[17] M. A. Eskan , R. Jotwani , T. Abe , et al., “The Leukocyte Integrin Antagonist Del‐1 Inhibits IL‐17‐Mediated Inflammatory Bone Loss,” Nature Immunology 13, no. 5 (2012): 465–473. 22447028 10.1038/ni.2260 PMC3330141

[18] A. L. Griffen , C. J. Beall , J. H. Campbell , et al., “Distinct and Complex Bacterial Profiles in Human Periodontitis and Health Revealed by 16S Pyrosequencing,” ISME Journal 6, no. 6 (2012): 1176–1185. 22170420 10.1038/ismej.2011.191 PMC3358035

[19] G. Hajishengallis , S. Liang , M. A. Payne , et al., “Low‐Abundance Biofilm Species Orchestrates Inflammatory Periodontal Disease Through the Commensal Microbiota and Complement,” Cell Host & Microbe 10, no. 5 (2011): 497–506. 22036469 10.1016/j.chom.2011.10.006 PMC3221781

[20] S. Offenbacher , S. P. Barros , D. W. Paquette , et al., “Gingival Transcriptome Patterns During Induction and Resolution of Experimental Gingivitis in Humans,” Journal of Periodontology 80, no. 12 (2009): 1963–1982. 19961380 10.1902/jop.2009.080645

[21] P. M. Preshaw and J. J. Taylor , “How Has Research Into Cytokine Interactions and Their Role in Driving Immune Responses Impacted Our Understanding of Periodontitis?,” Journal of Clinical Periodontology 38, no. Suppl 11 (2011): 60–84. 21323705 10.1111/j.1600-051X.2010.01671.x

[22] T. Yucel‐Lindberg and T. Bage , “Inflammatory Mediators in the Pathogenesis of Periodontitis,” Expert Reviews in Molecular Medicine 15 (2013): e7. 23915822 10.1017/erm.2013.8

[23] T. N. Crotti , A. A. Dharmapatni , E. Alias , and D. R. Haynes , “Osteoimmunology: Major and Costimulatory Pathway Expression Associated With Chronic Inflammatory Induced Bone Loss,” Journal of Immunology Research 2015 (2015): 281287. 26064999 10.1155/2015/281287 PMC4433696

[24] G. Hajishengallis , T. Abe , T. Maekawa , E. Hajishengallis , and J. D. Lambris , “Role of Complement in Host‐Microbe Homeostasis of the Periodontium,” Seminars in Immunology 25, no. 1 (2013): 65–72. 23684627 10.1016/j.smim.2013.04.004 PMC3706506

[25] A. Valverde , A. George , S. Nares , and A. R. Naqvi , “Emerging Therapeutic Strategies Targeting Bone Signaling Pathways in Periodontitis,” Journal of Periodontal Research 60, no. 2 (2025): 101–120. 39044454 10.1111/jre.13326 PMC11873684

[26] M. M. Osorio Parra , S. Elangovan , and C. T. Lee , “Specialized Pro‐Resolving Lipid Mediators in Experimental Periodontitis: A Systematic Review,” Oral Diseases 25, no. 5 (2019): 1265–1276. 30230662 10.1111/odi.12979

[27] C. N. Serhan , “Pro‐Resolving Lipid Mediators Are Leads for Resolution Physiology,” Nature 510, no. 7503 (2014): 92–101. 24899309 10.1038/nature13479 PMC4263681

[28] C. N. Serhan , N. Chiang , and J. Dalli , “New Pro‐Resolving n‐3 Mediators Bridge Resolution of Infectious Inflammation to Tissue Regeneration,” Molecular Aspects of Medicine 64 (2018): 1–17. 28802833 10.1016/j.mam.2017.08.002 PMC5832503

[29] C. N. Serhan and B. D. Levy , “Resolvins in Inflammation: Emergence of the Pro‐Resolving Superfamily of Mediators,” Journal of Clinical Investigation 128, no. 7 (2018): 2657–2669. 29757195 10.1172/JCI97943 PMC6025982

[30] O. Unlu , Z. Guney , and A. Kantarci , “Resolvin E1 and Maresin 1 Restore Senescence‐Induced Disruption of Human Periodontal Ligament Fibroblast Function,” Journal of Periodontology 96 (2025): 1168–1178. 40627765 10.1002/JPER.24-0565

[31] H. Hasturk , A. Kantarci , E. Goguet‐Surmenian , et al., “Resolvin E1 Regulates Inflammation at the Cellular and Tissue Level and Restores Tissue Homeostasis In Vivo,” Journal of Immunology 179, no. 10 (2007): 7021–7029. 10.4049/jimmunol.179.10.7021 17982093

[32] H. Hasturk , A. Kantarci , T. Ohira , et al., “RvE1 Protects From Local Inflammation and Osteoclast‐Mediated Bone Destruction in Periodontitis,” FASEB Journal 20, no. 2 (2006): 401–403. 16373400 10.1096/fj.05-4724fje

[33] C. T. Lee , R. Li , L. Zhu , et al., “Subgingival Microbiome and Specialized Pro‐Resolving Lipid Mediator Pathway Profiles Are Correlated in Periodontal Inflammation,” Frontiers in Immunology 12 (2021): 691216. 34177951 10.3389/fimmu.2021.691216 PMC8222734

[34] G. Mizraji , O. Heyman , T. E. van Dyke , and A. Wilensky , “Resolvin D2 Restrains Th1 Immunity and Prevents Alveolar Bone Loss in Murine Periodontitis,” Frontiers in Immunology 9 (2018): 785. 29922275 10.3389/fimmu.2018.00785 PMC5996935

[35] M. Sanz , D. Beighton , M. A. Curtis , et al., “Role of Microbial Biofilms in the Maintenance of Oral Health and in the Development of Dental Caries and Periodontal Diseases. Consensus Report of Group 1 of the Joint EFP/ORCA Workshop on the Boundaries Between Caries and Periodontal Disease,” Journal of Clinical Periodontology 44, no. Suppl 18 (2017): S5–S11. 28266109 10.1111/jcpe.12682

[36] M. Sanz , D. Herrera , M. Kebschull , et al., “Treatment of Stage I‐III Periodontitis‐The EFP S3 Level Clinical Practice Guideline,” Journal of Clinical Periodontology 47, no. Suppl 22 (2020): 4–60. 32383274 10.1111/jcpe.13290 PMC7891343

[37] V. Sahni and T. E. Van Dyke , “Immunomodulation of Periodontitis With SPMs,” Frontiers in Oral Health 4 (2023): 1288722. 37927821 10.3389/froh.2023.1288722 PMC10623003

[38] Y. D. Siddiqui , K. Omori , T. Ito , et al., “Resolvin D2 Induces Resolution of Periapical Inflammation and Promotes Healing of Periapical Lesions in Rat Periapical Periodontitis,” Frontiers in Immunology 10 (2019): 307. 30863409 10.3389/fimmu.2019.00307 PMC6399419

[39] B. S. Herrera , T. Ohira , L. Gao , et al., “An Endogenous Regulator of Inflammation, Resolvin E1, Modulates Osteoclast Differentiation and Bone Resorption,” British Journal of Pharmacology 155, no. 8 (2008): 1214–1223. 18806821 10.1038/bjp.2008.367 PMC2607210

[40] H. Hasturk , F. Schulte , M. Martins , et al., “Safety and Preliminary Efficacy of a Novel Host‐Modulatory Therapy for Reducing Gingival Inflammation,” Frontiers in Immunology 12 (2021): 704163. 34589083 10.3389/fimmu.2021.704163 PMC8475270

[41] H. Hasturk , G. Hajishengallis , and Forsyth Institute Center for C , “Phase IIa Clinical Trial of Complement C3 Inhibitor AMY‐101 in Adults With Periodontal Inflammation,” Journal of Clinical Investigation 131, no. 23 (2021): e152973. 34618684 10.1172/JCI152973 PMC8631591

[42] B. Alkaya , M. C. Haytac , M. Ozcan , et al., “Daily Probiotic Ayran Intake Reduces Gingival Inflammation: An Experimental Gingivitis Study,” Oral Health & Preventive Dentistry 22 (2024): 511–518. 39400083 10.3290/j.ohpd.b5784693 PMC11619859

[43] K. Lauwens , M. Saghi , P. J. Germonpre , et al., “Can we Combine Mouthrinses With Probiotics? An Evaluation of Their Compatibility and Combined Therapy on Oral Biofilms,” Journal of Periodontal Research (2025), 10.1111/jre.70033. 40928120

[44] B. Retamal‐Valdes , W. Teughels , L. M. Oliveira , et al., “Clinical, Microbiological, and Immunological Effects of Systemic Probiotics in Periodontal Treatment: Study Protocol for a Randomized Controlled Trial,” Trials 22, no. 1 (2021): 283. 33858486 10.1186/s13063-021-05246-0 PMC8048221

[45] W. van Holm , R. Carvalho , L. Delanghe , et al., “Antimicrobial Potential of Known and Novel Probiotics on In Vitro Periodontitis Biofilms,” NPJ Biofilms and Microbiomes 9, no. 1 (2023): 3. 36681674 10.1038/s41522-023-00370-y PMC9867767

[46] W. van Holm , K. Lauwens , P. de Wever , et al., “Probiotics for Oral Health: Do They Deliver What They Promise?,” Frontiers in Microbiology 14 (2023): 1219692. 37485503 10.3389/fmicb.2023.1219692 PMC10358723

[47] W. van Holm , N. Zayed , K. Lauwens , et al., “Oral Biofilm Composition, Dissemination to Keratinocytes, and Inflammatory Attenuation Depend on Probiotic and Synbiotic Strain Specificity,” Probiotics and Antimicrobial Proteins 17 (2024): 3041–3055. 38619794 10.1007/s12602-024-10253-z

[48] S. M. Gatej , V. Marino , R. Bright , et al., “Probiotic *Lactobacillus rhamnosus* GG Prevents Alveolar Bone Loss in a Mouse Model of Experimental Periodontitis,” Journal of Clinical Periodontology 45, no. 2 (2018): 204–212. 29121411 10.1111/jcpe.12838

[49] M. M. Invernici , F. A. C. Furlaneto , S. L. Salvador , et al., “ *Bifidobacterium animalis* Subsp Lactis HN019 Presents Antimicrobial Potential Against Periodontopathogens and Modulates the Immunological Response of Oral Mucosa in Periodontitis Patients,” PLoS One 15, no. 9 (2020): e0238425. 32960889 10.1371/journal.pone.0238425 PMC7508403

[50] M. S. Elburki , C. Rossa , M. R. Guimaraes , et al., “A Novel Chemically Modified Curcumin Reduces Severity of Experimental Periodontal Disease in Rats: Initial Observations,” Mediators of Inflammation 2014 (2014): 959471. 25104884 10.1155/2014/959471 PMC4101223

[51] N. A. R. Fernandes , A. C. Camilli , L. A. G. Maldonado , et al., “Chalcone T4, a Novel Chalconic Compound, Inhibits Inflammatory Bone Resorption In Vivo and Suppresses Osteoclastogenesis In Vitro,” Journal of Periodontal Research 56, no. 3 (2021): 569–578. 33641160 10.1111/jre.12857

[52] M. R. Guimaraes , L. S. Coimbra , S. G. de Aquino , L. C. Spolidorio , K. L. Kirkwood , and C. Rossa, Jr. , “Potent Anti‐Inflammatory Effects of Systemically Administered Curcumin Modulate Periodontal Disease In Vivo,” Journal of Periodontal Research 46, no. 2 (2011): 269–279. 21306385 10.1111/j.1600-0765.2010.01342.x PMC3086370

[53] A. L. R. Pavanelli , S. M. Vieira , C. C. Marcantonio , et al., “Anti‐Inflammatory and Antiresorptive Activities of Tanshinone‐IIA Mitigate Alveolar Bone Destruction in Mice With Experimental Periodontitis,” Journal of Periodontology 96 (2025): 1138–1153. 40663013 10.1002/JPER.24-0618

[54] R. S. de Molon , “Therapeutic Potential of Tanshinones in Osteolytic Diseases: From Molecular and Cellular Pathways to Preclinical Models,” Dentistry Journal 13, no. 7 (2025): 309. 40710154 10.3390/dj13070309 PMC12293886

[55] Y. Zhu , A. Ali , G. Mulinari Dos Santos , et al., “A Chitosan‐Based Hydrogel to Modulate Immune Cells and Promote Periodontitis Healing in the High‐Fat Diet‐Induced Periodontitis Rat Model,” Acta Biomaterialia 200 (2025): 452–463. 40379118 10.1016/j.actbio.2025.05.034

[56] N. Da Ponte Leguizamon , R. S. de Molon , G. Coletto‐Nunes , et al., “Phytocystatin CsinCPI‐2 Reduces Osteoclastogenesis and Alveolar Bone Loss,” Journal of Dental Research 101, no. 2 (2022): 216–225. 34328027 10.1177/00220345211027811

[57] E. G. Eltay and T. van Dyke , “Resolution of Inflammation in Oral Diseases,” Pharmacology & Therapeutics 247 (2023): 108453. 37244405 10.1016/j.pharmthera.2023.108453

[58] J. Panezai and T. E. van Dyke , “Resolution of Inflammation: Intervention Strategies and Future Applications,” Toxicology and Applied Pharmacology 449 (2022): 116089. 35644268 10.1016/j.taap.2022.116089

[59] G. G. Guarenghi , P. A. T. Ribas , R. M. Ferro , et al., “App‐Guided Exercise Improves Periodontal Status in Periodontitis Treatment – A Pilot Randomized Clinical Trial,” Pesquisa Brasileira Em Odontopediatria e Clínica Integrada 26 (2025): e240136.

[60] V. G. Garcia , D. M. J. Miessi , T. E. da Rocha , et al., “Shedding Light on the Therapeutic Efficiency of Oxygen‐Releasing Gel and Photodynamic Therapy as Adjuvants in the Treatment of Experimental Periodontitis,” Photobiomodulation, Photomedicine, and Laser Surgery 43, no. 4 (2025): 159–172. 40095942 10.1089/photob.2024.0083

[61] G. E. Oliveira , D. da Silva Barbirato , B. S. de Menezes , et al., “Exploring the Impact of Biological Agents on Protecting Against Experimental Periodontitis: A Systematic Review of Animal‐Based Studies,” BioMed Research International 2024 (2024): 1716735. 39654845 10.1155/bmri/1716735 PMC11628168

[62] A. L. R. Pavanelli , B. S. de Menezes , E. B. B. Pereira , F. A. de Souza Morais , J. A. Cirelli , and R. S. de Molon , “Pharmacological Therapies for the Management of Inflammatory Bone Resorption in Periodontal Disease: A Review of Preclinical Studies,” BioMed Research International 2022 (2022): 5832009. 35547360 10.1155/2022/5832009 PMC9085331

[63] R. S. de Molon , E. D. de Avila , A. V. Boas Nogueira , et al., “Evaluation of the Host Response in Various Models of Induced Periodontal Disease in Mice,” Journal of Periodontology 85, no. 3 (2014): 465–477. 23805811 10.1902/jop.2013.130225

[64] R. S. de Molon , E. D. de Avila , and J. A. Cirelli , “Host Responses Induced by Different Animal Models of Periodontal Disease: A Literature Review,” Journal of Investigative and Clinical Dentistry 4, no. 4 (2013): 211–218. 23188588 10.1111/jicd.12018

[65] R. S. de Molon , V. I. Mascarenhas , E. D. de Avila , et al., “Long‐Term Evaluation of Oral Gavage With Periodontopathogens or Ligature Induction of Experimental Periodontal Disease in Mice,” Clinical Oral Investigations 20, no. 6 (2016): 1203–1216. 26411857 10.1007/s00784-015-1607-0

[66] R. S. de Molon , C. H. Park , Q. Jin , J. Sugai , and J. A. Cirelli , “Characterization of Ligature‐Induced Experimental Periodontitis,” Microscopy Research and Technique 81, no. 12 (2018): 1412–1421. 30351474 10.1002/jemt.23101

[67] D. T. Graves , D. Fine , Y. T. Teng , T. E. van Dyke , and G. Hajishengallis , “The Use of Rodent Models to Investigate Host‐Bacteria Interactions Related to Periodontal Diseases,” Journal of Clinical Periodontology 35, no. 2 (2008): 89–105. 18199146 10.1111/j.1600-051X.2007.01172.x PMC2649707

[68] D. T. Graves , J. Kang , O. Andriankaja , K. Wada , and C. Rossa , “Animal Models to Study Host‐Bacteria Interactions Involved in Periodontitis,” Frontiers of Oral Biology 15 (2012): 117–132. 22142960 10.1159/000329675 PMC3766715

[69] T. Abe and G. Hajishengallis , “Optimization of the Ligature‐Induced Periodontitis Model in Mice,” Journal of Immunological Methods 394, no. 1–2 (2013): 49–54. 23672778 10.1016/j.jim.2013.05.002 PMC3707981

[70] C. Rojas , M. P. Garcia , A. F. Polanco , et al., “Humanized Mouse Models for the Study of Periodontitis: An Opportunity to Elucidate Unresolved Aspects of Its Immunopathogenesis and Analyze New Immunotherapeutic Strategies,” Frontiers in Immunology 12 (2021): 663328. 34220811 10.3389/fimmu.2021.663328 PMC8248545

[71] M. Di Stefano , A. Polizzi , S. Santonocito , A. Romano , T. Lombardi , and G. Isola , “Impact of Oral Microbiome in Periodontal Health and Periodontitis: A Critical Review on Prevention and Treatment,” International Journal of Molecular Sciences 23, no. 9 (2022): 5142. 35563531 10.3390/ijms23095142 PMC9103139

[72] A. V. Nogueira , R. S. de Molon , M. Nokhbehsaim , J. Deschner , and J. A. Cirelli , “Contribution of Biomechanical Forces to Inflammation‐Induced Bone Resorption,” Journal of Clinical Periodontology 44, no. 1 (2017): 31–41. 27716969 10.1111/jcpe.12636

[73] J. Cavagni , I. C. de Macedo , E. J. Gaio , et al., “Obesity and Hyperlipidemia Modulate Alveolar Bone Loss in Wistar Rats,” Journal of Periodontology 87, no. 2 (2016): e9–e17. 26376945 10.1902/jop.2015.150330

[74] S. Macari , M. F. M. Madeira , I. L. A. Lima , et al., “ST2 Regulates Bone Loss in a Site‐Dependent and Estrogen‐Dependent Manner,” Journal of Cellular Biochemistry 119, no. 10 (2018): 8511–8521. 30011081 10.1002/jcb.27080

[75] B. R. Silva , M. A. R. Hidalgo , R. C. L. Silva , et al., “Establishing a Dual Murine Model to Explore the Interactions Between Diabetes and Periodontitis in Mice,” International Journal of Molecular Sciences 26, no. 12 (2025): 5611. 40565075 10.3390/ijms26125611 PMC12192739

[76] F. Khuda , B. Baharin , N. N. M. Anuar , B. S. F. Satimin , and N. S. Nasruddin , “Effective Modalities of Periodontitis Induction in Rat Model,” Journal of Veterinary Dentistry 41, no. 1 (2024): 49–57. 37259505 10.1177/08987564231178459

[77] J. G. Messer , S. La , D. E. Kipp , et al., “Diet‐Induced Generalized Periodontitis in Lewis Rats,” Comparative Medicine 69, no. 5 (2019): 384–400. 31575381 10.30802/AALAS-CM-18-000113 PMC6807724

[78] H. S. Oz and D. A. Puleo , “Animal Models for Periodontal Disease,” Journal of Biomedicine and Biotechnology 2011 (2011): 754857. 21331345 10.1155/2011/754857 PMC3038839

[79] M. Munar‐Bestard , O. Villa , M. D. M. Ferra‐Canellas , J. M. Ramis , and M. Monjo , “Induction of Periodontitis via a Combination of Ligature and Lipopolysaccharide Injection in a Rat Model,” Journal of Visualized Experiments (2023): 192, 10.3791/64842. 36876950

[80] M. Tariq , Z. Iqbal , J. Ali , et al., “Treatment Modalities and Evaluation Models for Periodontitis,” International Journal of Pharmaceutical Investigation 2, no. 3 (2012): 106–122. 23373002 10.4103/2230-973X.104394 PMC3555006

[81] Y. Wei , Y. Deng , S. Ma , et al., “Local Drug Delivery Systems as Therapeutic Strategies Against Periodontitis: A Systematic Review,” Journal of Controlled Release 333 (2021): 269–282. 33798664 10.1016/j.jconrel.2021.03.041

[82] B. Yilmaz , Y. Yildirim , N. Yakar , G. Ozdemir , A. Kantarci , and G. Emingil , “Dual‐Drug Carboxymethyl Chitosan Hydrogel: Development, Characterization, and In Vitro Evaluation for Periodontal Therapy,” Carbohydrate Polymers 363 (2025): 123726. 40441835 10.1016/j.carbpol.2025.123726

[83] D. I. Lewis , “Animal Experimentation: Implementation and Application of the 3Rs,” Emerging Topics in Life Sciences 3, no. 6 (2019): 675–679. 32915219 10.1042/ETLS20190061

[84] D. Calabrese , “Management of Cardiovascular Disease in Chronic Kidney Disease: Implications for Managed Care,” American Journal of Managed Care 17, no. Suppl 15 (2011): S412–S418. 22214476

[85] X. Struillou , H. Boutigny , A. Soueidan , and P. Layrolle , “Experimental Animal Models in Periodontology: A Review,” Open Dentistry Journal 4 (2010): 37–47. 20556202 10.2174/1874210601004010037 PMC2885595

[86] R. C. Page and H. E. Schroeder , “Pathogenesis of Inflammatory Periodontal Disease. A Summary of Current Work,” Laboratory Investigation 34, no. 3 (1976): 235–249. 765622

[87] S. S. Socransky and A. D. Haffajee , “Microbial Mechanisms in the Pathogenesis of Destructive Periodontal Diseases: A Critical Assessment,” Journal of Periodontal Research 26, no. 3 Pt 2 (1991): 195–212. 10.1111/j.1600-0765.1991.tb01646.x 1831843

[88] L. M. Golub and H. M. Lee , “Periodontal Therapeutics: Current Host‐Modulation Agents and Future Directions,” Periodontology 2000 82, no. 1 (2020): 186–204. 31850625 10.1111/prd.12315 PMC6973248

[89] S. Nyman , J. Lindhe , T. Karring , and H. Rylander , “New Attachment Following Surgical Treatment of Human Periodontal Disease,” Journal of Clinical Periodontology 9, no. 4 (1982): 290–296. 6964676 10.1111/j.1600-051x.1982.tb02095.x

[90] G. Hajishengallis and R. J. Lamont , “Metabolic Nuclear Receptors in Periodontal Host‐Microbe Interactions and Inflammation,” Molecular Oral Microbiology 32, no. 6 (2017): 443–445. 28984043 10.1111/omi.12198

[91] G. Hajishengallis and R. J. Lamont , “Polymicrobial Communities in Periodontal Disease: Their Quasi‐Organismal Nature and Dialogue With the Host,” Periodontology 2000 86, no. 1 (2021): 210–230. 33690950 10.1111/prd.12371 PMC8957750

[92] R. J. Lamont , H. Koo , and G. Hajishengallis , “The Oral Microbiota: Dynamic Communities and Host Interactions,” Nature Reviews. Microbiology 16, no. 12 (2018): 745–759. 30301974 10.1038/s41579-018-0089-x PMC6278837

[93] C. Zenobia , X. L. Luo , A. Hashim , et al., “Commensal Bacteria‐Dependent Select Expression of CXCL2 Contributes to Periodontal Tissue Homeostasis,” Cellular Microbiology 15, no. 8 (2013): 1419–1426. 23433011 10.1111/cmi.12127 PMC3711967

[94] B. G. Loos and T. E. Van Dyke , “The Role of Inflammation and Genetics in Periodontal Disease,” Periodontology 2000 83, no. 1 (2020): 26–39. 32385877 10.1111/prd.12297 PMC7319430

[95] G. Hajishengallis , R. P. Darveau , and M. A. Curtis , “The Keystone‐Pathogen Hypothesis,” Nature Reviews. Microbiology 10, no. 10 (2012): 717–725. 22941505 10.1038/nrmicro2873 PMC3498498

[96] G. Hajishengallis and R. J. Lamont , “Breaking Bad: Manipulation of the Host Response by *Porphyromonas gingivalis* ,” European Journal of Immunology 44, no. 2 (2014): 328–338. 24338806 10.1002/eji.201344202 PMC3925422

[97] I. Olsen , J. D. Lambris , and G. Hajishengallis , “ *Porphyromonas gingivalis* Disturbs Host‐Commensal Homeostasis by Changing Complement Function,” Journal of Oral Microbiology 9, no. 1 (2017): 1340085. 28748042 10.1080/20002297.2017.1340085 PMC5508361

[98] G. Hajishengallis , T. Chavakis , and J. D. Lambris , “Current Understanding of Periodontal Disease Pathogenesis and Targets for Host‐Modulation Therapy,” Periodontology 2000 84, no. 1 (2020): 14–34. 32844416 10.1111/prd.12331 PMC7457922

[99] S. Li , W. Zeng , G. Liu , J. Zang , and X. Yu , “Evaluation of Morphological, Histological, and Immune‐Related Cellular Changes in Ligature‐Induced Experimental Periodontitis in Mice,” Journal of Dental Sciences 18, no. 4 (2023): 1716–1722. 37799858 10.1016/j.jds.2023.01.002 PMC10547956

[100] P. Lin , H. Niimi , Y. Ohsugi , et al., “Application of Ligature‐Induced Periodontitis in Mice to Explore the Molecular Mechanism of Periodontal Disease,” International Journal of Molecular Sciences 22, no. 16 (2021): 8900. 34445604 10.3390/ijms22168900 PMC8396362

[101] P. M. Preshaw , “Host Modulation Therapy With Anti‐Inflammatory Agents,” Periodontology 2000 76, no. 1 (2018): 131–149. 29193331 10.1111/prd.12148

[102] L. M. Sedghi , M. Bacino , and Y. L. Kapila , “Periodontal Disease: The Good, the Bad, and the Unknown,” Frontiers in Cellular and Infection Microbiology 11 (2021): 766944. 34950607 10.3389/fcimb.2021.766944 PMC8688827

[103] L. S. Coimbra , J. P. Steffens , C. Rossa, Jr. , D. T. Graves , and L. C. Spolidorio , “Clopidogrel Enhances Periodontal Repair in Rats Through Decreased Inflammation,” Journal of Clinical Periodontology 41, no. 3 (2014): 295–302. 24433307 10.1111/jcpe.12203 PMC3952018

[104] M. R. Guimaraes‐Stabili , S. G. de Aquino , F. Almeida Curylofo , et al., “Systemic Administration of Curcumin or Piperine Enhances the Periodontal Repair: A Preliminary Study in Rats,” Clinical Oral Investigations 23, no. 8 (2019): 3297–3306. 30498979 10.1007/s00784-018-2755-9

[105] L. C. Spolidorio , P. D. Lucas , J. P. Steffens , et al., “Influence of Parstatin on Experimental Periodontal Disease and Repair in Rats,” Journal of Periodontology 85, no. 9 (2014): 1266–1274. 24410294 10.1902/jop.2014.130619

[106] J. P. Steffens , L. C. L. Santana , J. C. P. Pitombo , et al., “The Role of Androgens on Periodontal Repair in Female Rats,” Journal of Periodontology 89, no. 4 (2018): 486–495. 29683499 10.1002/JPER.17-0435

[107] J. V. S. Roth , P. A. T. Ribas , H. K. Takarada , et al., “Local and Systemic Characterization of Inflammatory Profile in Long‐Term Ligature‐Induced Periodontitis and Repair in Rats,” Brazilian Journal of Oral Sciences 24 (2025): e258463.

[108] J. A. C. Souza , F. A. C. Magalhaes , G. Oliveira , J. A. Zuanon , and P. P. C. Souza , “Pam2CSK4 (TLR2 Agonist) Induces Periodontal Destruction in Mice,” Brazilian Oral Research 34 (2020): e012. 32049112 10.1590/1807-3107bor-2020.vol34.0012

[109] N. O. Bertolini , G. J. S. Pereira , V. O. Silva , et al., “Voluntary Physical Activity Mitigates Alveolar Bone Loss in Mice With Ligature‐Induced Experimental Periodontitis,” Archives of Oral Biology 140 (2022): 105451. 35617755 10.1016/j.archoralbio.2022.105451

[110] V. G. Garcia , L. R. Knoll , M. Longo , et al., “Effect of the Probiotic *Saccharomyces cerevisiae* on Ligature‐Induced Periodontitis in Rats,” Journal of Periodontal Research 51, no. 1 (2016): 26–37. 25918871 10.1111/jre.12274

[111] T. Kwon , I. B. Lamster , and L. Levin , “Current Concepts in the Management of Periodontitis,” International Dental Journal 71, no. 6 (2021): 462–476. 34839889 10.1111/idj.12630 PMC9275292

[112] M. C. Basil and B. D. Levy , “Specialized Pro‐Resolving Mediators: Endogenous Regulators of Infection and Inflammation,” Nature Reviews. Immunology 16, no. 1 (2016): 51–67. 10.1038/nri.2015.4 PMC5242505 26688348

[113] G. Fredman and C. N. Serhan , “Specialized Proresolving Mediator Targets for RvE1 and RvD1 in Peripheral Blood and Mechanisms of Resolution,” Biochemical Journal 437, no. 2 (2011): 185–197. 21711247 10.1042/BJ20110327 PMC3133883

[114] J. L. Cash , L. V. Norling , and M. Perretti , “Resolution of Inflammation: Targeting GPCRs That Interact With Lipids and Peptides,” Drug Discovery Today 19, no. 8 (2014): 1186–1192. 24993159 10.1016/j.drudis.2014.06.023 PMC4154450

[115] N. Chiang , S. Libreros , P. C. Norris , X. de la Rosa , and C. N. Serhan , “Maresin 1 Activates LGR6 Receptor Promoting Phagocyte Immunoresolvent Functions,” Journal of Clinical Investigation 129, no. 12 (2019): 5294–5311. 31657786 10.1172/JCI129448 PMC6877300

[116] S. Krishnamoorthy , A. Recchiuti , N. Chiang , G. Fredman , and C. N. Serhan , “Resolvin D1 Receptor Stereoselectivity and Regulation of Inflammation and Proresolving microRNAs,” American Journal of Pathology 180, no. 5 (2012): 2018–2027. 22449948 10.1016/j.ajpath.2012.01.028 PMC3349829

[117] T. E. van Dyke and C. Sima , “Understanding Resolution of Inflammation in Periodontal Diseases: Is Chronic Inflammatory Periodontitis a Failure to Resolve?,” Periodontology 2000 82, no. 1 (2020): 205–213. 31850636 10.1111/prd.12317

[118] C. F. Araujo , N. Andere , N. C. Castro Dos Santos , et al., “Omega‐3 and Aspirin in the Nonsurgical Treatment of Grade C Periodontitis: A Randomized Clinical Trial,” Journal of Periodontology 96, no. 8 (2025): 881–893. 39950354 10.1002/JPER.24-0322

[119] N. C. Castro Dos Santos , N. Andere , C. F. Araujo , et al., “Omega‐3 PUFA and Aspirin as Adjuncts to Periodontal Debridement in Patients With Periodontitis and Type 2 Diabetes Mellitus: Randomized Clinical Trial,” Journal of Periodontology 91, no. 10 (2020): 1318–1327. 32103495 10.1002/JPER.19-0613 PMC7483813

[120] I. Keskiner , I. Saygun , V. Bal , M. Serdar , and A. Kantarci , “Dietary Supplementation With Low‐Dose Omega‐3 Fatty Acids Reduces Salivary Tumor Necrosis Factor‐Alpha Levels in Patients With Chronic Periodontitis: A Randomized Controlled Clinical Study,” Journal of Periodontal Research 52, no. 4 (2017): 695–703. 28177133 10.1111/jre.12434

[121] M. A. Anwar , G. A. Sayed , D. M. Hal , et al., “Herbal Remedies for Oral and Dental Health: A Comprehensive Review of Their Multifaceted Mechanisms Including Antimicrobial, Anti‐Inflammatory, and Antioxidant Pathways,” Inflammopharmacology 33, no. 3 (2025): 1085–1160. 39907951 10.1007/s10787-024-01631-8 PMC11914039

[122] G. Malcangi , A. M. Inchingolo , L. Casamassima , et al., “Effectiveness of Herbal Medicines With Anti‐Inflammatory, Antimicrobial, and Antioxidant Properties in Improving Oral Health and Treating Gingivitis and Periodontitis: A Systematic Review,” Nutrients 17, no. 5 (2025): 762. 40077632 10.3390/nu17050762 PMC11901544

[123] C. J. Xiao , X. J. Yu , J. L. Xie , S. Liu , and S. Li , “Protective Effect and Related Mechanisms of Curcumin in Rat Experimental Periodontitis,” Head & Face Medicine 14, no. 1 (2018): 12. 30115081 10.1186/s13005-018-0169-1 PMC6097422

[124] R. R. Hosadurga , S. N. Rao , J. Jose , N. C. Rompicharla , M. Shakil , and R. Shashidhara , “Evaluation of the Efficacy of 2% Curcumin Gel in the Treatment of Experimental Periodontitis,” Pharmacognosy Research 6, no. 4 (2014): 326–333. 25276071 10.4103/0974-8490.138287 PMC4166822

[125] H. B. Samal , L. Boyeena , S. A. Sreenivas , and I. Jogamaya Das , “Design, Characterization and Clinical Evaluation of Curcumin Dental Film for the Treatment of Periodontitis,” Drug Delivery Letters 11, no. 1 (2020): 81–95.

[126] Y. Wang , H. Lin , W. Huang , et al., “Curcumin Attenuates Periodontal Injury via Inhibiting Ferroptosis of Ligature‐Induced Periodontitis in Mice,” International Journal of Molecular Sciences 24, no. 12 (2023): 9835. 37372983 10.3390/ijms24129835 PMC10298010

[127] T. Minagawa , T. Okui , N. Takahashi , et al., “Resveratrol Suppresses the Inflammatory Responses of Human Gingival Epithelial Cells in a SIRT1 Independent Manner,” Journal of Periodontal Research 50, no. 5 (2015): 586–593. 25312218 10.1111/jre.12238

[128] G. Bhattarai , S. B. Poudel , S. H. Kook , and J. C. Lee , “Resveratrol Prevents Alveolar Bone Loss in an Experimental Rat Model of Periodontitis,” Acta Biomaterialia 29 (2016): 398–408. 26497626 10.1016/j.actbio.2015.10.031

[129] R. Conte , A. Valentino , F. Sepe , et al., “Resveratrol‐Loaded Solid Lipid Nanoparticles Reinforced Hyaluronic Hydrogel: Multitarget Strategy for the Treatment of Diabetes‐Related Periodontitis,” Biomedicine 13, no. 5 (2025): 1059. 10.3390/biomedicines13051059 PMC12108562 40426886

[130] L. Zhen , D. S. Fan , Y. Zhang , X. M. Cao , and L. M. Wang , “Resveratrol Ameliorates Experimental Periodontitis in Diabetic Mice Through Negative Regulation of TLR4 Signaling,” Acta Pharmacologica Sinica 36, no. 2 (2015): 221–228. 25530164 10.1038/aps.2014.131 PMC4326790

[131] T. M. Barber , S. Kabisch , H. S. Randeva , A. F. H. Pfeiffer , and M. O. Weickert , “Implications of Resveratrol in Obesity and Insulin Resistance: A State‐Of‐The‐Art Review,” Nutrients 14, no. 14 (2022): 2870. 35889827 10.3390/nu14142870 PMC9320680

[132] I. M. Kapetanovic , M. Muzzio , Z. Huang , T. N. Thompson , and D. L. McCormick , “Pharmacokinetics, Oral Bioavailability, and Metabolic Profile of Resveratrol and Its Dimethylether Analog, Pterostilbene, in Rats,” Cancer Chemotherapy and Pharmacology 68, no. 3 (2011): 593–601. 21116625 10.1007/s00280-010-1525-4 PMC3090701

[133] A. Z. Javid , R. Hormoznejad , H. A. Yousefimanesh , M. H. Haghighi‐Zadeh , and M. Zakerkish , “Impact of Resveratrol Supplementation on Inflammatory, Antioxidant, and Periodontal Markers in Type 2 Diabetic Patients With Chronic Periodontitis,” Diabetes & Metabolic Syndrome 13, no. 4 (2019): 2769–2774. 31405706 10.1016/j.dsx.2019.07.042

[134] V. H. Lucchesi , A. P. O. Giorgetti , M. G. Correa , et al., “Impact of Systemic Resveratrol on Non‐Surgical Periodontal Treatment of Smokers: A 12‐Month Randomized Clinical Trial,” Clinical Oral Investigations 29, no. 9 (2025): 428. 40884630 10.1007/s00784-025-06517-9

[135] S. Nikniaz , F. Vaziri , and R. Mansouri , “Impact of Resveratrol Supplementation on Clinical Parameters and Inflammatory Markers in Patients With Chronic Periodontitis: A Randomized Clinical Trail,” BMC Oral Health 23, no. 1 (2023): 177. 36973728 10.1186/s12903-023-02877-4 PMC10045616

[136] H. Huangfu , S. Du , H. Zhang , et al., “Facile Engineering of Resveratrol Nanoparticles Loaded With 20(S)‐Protopanaxadiol for the Treatment of Periodontitis by Regulating the Macrophage Phenotype,” Nanoscale 15, no. 17 (2023): 7894–7908. 37060139 10.1039/d2nr06452a

[137] S. N. Gottumukkala , S. Koneru , S. Mannem , and N. Mandalapu , “Effectiveness of Sub Gingival Irrigation of an Indigenous 1% Curcumin Solution on Clinical and Microbiological Parameters in Chronic Periodontitis Patients: A Pilot Randomized Clinical Trial,” Contemporary Clinical Dentistry 4, no. 2 (2013): 186–191. 24015007 10.4103/0976-237X.114874 PMC3757880

[138] K. V. Raghava , K. P. Sistla , S. J. Narayan , U. Yadalam , A. Bose , and K. Mitra , “Efficacy of Curcumin as an Adjunct to Scaling and Root Planing in Chronic Periodontitis Patients: A Randomized Controlled Clinical Trial,” Journal of Contemporary Dental Practice 20, no. 7 (2019): 842–846. 31597806

[139] S. Sharath , D. G. Kamath , and N. Shenoy , “Comparative Evaluation of Curcumin Gel With Diode Laser (970 Nm) Versus Diode Laser Alone for Pocket Depth Reduction in Chronic Localised Periodontitis ‐ A Split‐Mouth Randomized Clinical Trial,” Journal of Lasers in Medical Sciences 16 (2025): e8. 40666251 10.34172/jlms.2025.08 PMC12260991

[140] S. Terby , M. Shereef , V. Ramanarayanan , and B. Balakrishnan , “The Effect of Curcumin as an Adjunct in the Treatment of Chronic Periodontitis: A Systematic Review and Meta‐Analysis,” Saudi Dental Journal 33, no. 7 (2021): 375–385. 34803277 10.1016/j.sdentj.2021.07.008 PMC8589622

[141] Y. Zhang , L. Huang , D. Mazurel , H. Zheng , J. Yang , and D. Deng , “Clinical Efficacy of Curcumin Versus Chlorhexidine as an Adjunct to Scaling and Root Planing for the Treatment of Periodontitis: A Systematic Review and Meta‐Analysis,” Phytotherapy Research 35, no. 11 (2021): 5980–5991. 34216058 10.1002/ptr.7208

[142] R. S. Cardoso , M. R. Messora , P. H. F. Silva , et al., “Effects of *Bifidobacterium animalis* Subsp. Lactis HN019 on Ligature‐Induced Periodontitis in Rats With Experimental Rheumatoid Arthritis,” Beneficial Microbes 11, no. 1 (2020): 33–46. 32066256 10.3920/BM2019.0038

[143] R. L. Lucateli , P. H. F. Silva , S. L. Salvador , et al., “Probiotics Enhance Alveolar Bone Microarchitecture, Intestinal Morphology and Estradiol Levels in Osteoporotic Animals,” Journal of Periodontal Research 59, no. 4 (2024): 758–770. 38699835 10.1111/jre.13256

[144] D. N. A. Silva , N. T. S. Cruz , A. A. Martins , et al., “Probiotic *Lactobacillus rhamnosus* EM1107 Prevents Hyperglycemia, Alveolar Bone Loss, and Inflammation in a Rat Model of Diabetes and Periodontitis,” Journal of Periodontology 94, no. 3 (2023): 376–388. 36322996 10.1002/JPER.22-0262

[145] B. Alkaya , I. Laleman , S. Keceli , O. Ozcelik , M. Cenk Haytac , and W. Teughels , “Clinical Effects of Probiotics Containing Bacillus Species on Gingivitis: A Pilot Randomized Controlled Trial,” Journal of Periodontal Research 52, no. 3 (2017): 497–504. 27859252 10.1111/jre.12415

[146] B. E. Kuru , I. Laleman , T. Yalnizoglu , L. Kuru , and W. Teughels , “The Influence of a *Bifidobacterium animalis* Probiotic on Gingival Health: A Randomized Controlled Clinical Trial,” Journal of Periodontology 88, no. 11 (2017): 1115–1123. 28753102 10.1902/jop.2017.170213

[147] I. Laleman , M. Pauwels , M. Quirynen , and W. Teughels , “A Dual‐Strain Lactobacilli Reuteri Probiotic Improves the Treatment of Residual Pockets: A Randomized Controlled Clinical Trial,” Journal of Clinical Periodontology 47, no. 1 (2020): 43–53. 31520543 10.1111/jcpe.13198 PMC6973056

[148] I. Laleman , E. Yilmaz , O. Ozcelik , et al., “The Effect of a Streptococci Containing Probiotic in Periodontal Therapy: A Randomized Controlled Trial,” Journal of Clinical Periodontology 42, no. 11 (2015): 1032–1041. 26427036 10.1111/jcpe.12464

[149] N. S. Abdul , L. G. Odeh , A. A. Alenazi , J. A. Alzahrani , A. T. Almutib , and C. Soman , “Probiotics in the Prevention and Treatment of Periodontal Diseases: A Systematic Review,” Journal of Pharmacy & Bioallied Sciences 16, no. Suppl 4 (2024): S3302–S3307. 39927018 10.4103/jpbs.jpbs_681_24 PMC11804984

[150] C. Benavides‐Reyes , I. Cabello , A. Magan‐Fernandez , M. Rodriguez‐Barranco , S. N. Usta , and F. Mesa , “Clinical Effects of Probiotics on the Treatment of Gingivitis and Periodontitis: A Systematic Review and Meta‐Analysis,” BMC Oral Health 25, no. 1 (2025): 490. 40186219 10.1186/s12903-025-05888-5 PMC11971800

[151] C. Mendonca , D. Marques , J. Silveira , J. Marques , R. F. de Souza , and A. Mata , “Effects of Probiotic Therapy on Periodontal and Peri‐Implant Treatments: An Umbrella Review,” JDR Clinical & Translational Research 10, no. 3 (2025): 246–268. 39508204 10.1177/23800844241240474 PMC12166148

[152] C. D. Mendonca , A. Mata , L. F. R. Azevedo , J. F. Marques , J. M. L. Silveira , and D. Marques , “Probiotics in the Non‐Surgical Treatment of Periodontitis: A Systematic Review and Network Meta‐Analysis,” BMC Oral Health 24, no. 1 (2024): 1224. 39407177 10.1186/s12903-024-05027-6 PMC11481756

[153] J. Lu , X. He , T. Du , and D. Fu , “Clinical Effects of *Lactobacillus reuteri* on Gingival Inflammation and Alveolar Bone Loss in Periodontitis,” Oral Health & Preventive Dentistry 23 (2025): 585–591. 41026095 10.3290/j.ohpd.c_2289 PMC12532032

[154] F. C. Yilmaz and N. C. Gorgin , “The Role of Probiotics and Dietary Interventions in the Treatment of Periodontitis: A Pilot Randomized Controlled Clinical Trial,” BMC Oral Health 25, no. 1 (2025): 1287. 40745652 10.1186/s12903-025-06510-4 PMC12315224

[155] P. Huo , L. Deng , J. Lu , P. Kan , R. Jing , and L. J. Luo , “The Impact of Limosilactobacillus Reuteri in Combination With Non‐Surgical Periodontal Therapy on Periodontal Clinical Parameters and Salivary and Subgingival Microbiota Composition in Individuals With Stage III‐IV Periodontitis: A Randomized Controlled Trial,” BMC Oral Health 25, no. 1 (2025): 759. 40405209 10.1186/s12903-025-06084-1 PMC12096711

[156] M. A. N. Jardini , J. F. Pedroso , C. L. Ferreira , et al., “Effect of Adjuvant Probiotic Therapy ( *Lactobacillus reuteri* ) in the Treatment of Periodontitis Associated With Diabetes Mellitus: Clinical, Controlled, and Randomized Study,” Clinical Oral Investigations 28, no. 1 (2024): 80. 38183505 10.1007/s00784-023-05441-0

[157] T. C. S. Ramos , M. L. V. Boas , C. M. M. Nunes , et al., “Effect of Systemic Antibiotic and Probiotic Therapies as Adjuvant Treatments of Subgingival Instrumentation for Periodontitis: A Randomized Controlled Clinical Study,” Journal of Applied Oral Science 30 (2022): e20210583. 35319669 10.1590/1678-7757-2021-0583 PMC8963390

[158] Y. L. de Almeida Silva Levi , M. C. Ribeiro , P. H. F. Silva , et al., “Effects of Oral Administration of *Bifidobacterium animalis* Subsp. Lactis HN019 on the Treatment of Plaque‐Induced Generalized Gingivitis,” Clinical Oral Investigations 27, no. 1 (2023): 387–398. 36305963 10.1007/s00784-022-04744-y PMC9614197

[159] G. Hajishengallis , T. Kajikawa , E. Hajishengallis , et al., “Complement‐Dependent Mechanisms and Interventions in Periodontal Disease,” Frontiers in Immunology 10 (2019): 406. 30915073 10.3389/fimmu.2019.00406 PMC6422998

[160] J. Dernedde , A. Rausch , M. Weinhart , et al., “Dendritic Polyglycerol Sulfates as Multivalent Inhibitors of Inflammation,” Proceedings of the National Academy of Sciences of the United States of America 107, no. 46 (2010): 19679–19684. 21041668 10.1073/pnas.1003103107 PMC2993387

[161] V. G. Garcia , L. A. Fernandes , V. C. Macarini , et al., “Treatment of Experimental Periodontal Disease With Antimicrobial Photodynamic Therapy in Nicotine‐Modified Rats,” Journal of Clinical Periodontology 38, no. 12 (2011): 1106–1114. 22092666 10.1111/j.1600-051X.2011.01785.x

[162] M. A. A. Nuernberg , M. Wainwright , D. M. J. Miessi , et al., “Effects of Butyl Toluidine Blue Photosensitizer on Antimicrobial Photodynamic Therapy for Experimental Periodontitis Treatment in Rats,” Photodiagnosis and Photodynamic Therapy 31 (2020): 101868. 32526374 10.1016/j.pdpdt.2020.101868

[163] A. P. Santana , D. M. Cunha , R. D. Piazza , et al., “Butyl Toluidine Blue as a Photosensitizer for Antimicrobial Photodynamic Therapy on Titanium‐Associated Biofilms,” Journal of Photochemistry and Photobiology, B: Biology 273 (2025): 113274. 41175422 10.1016/j.jphotobiol.2025.113274

[164] J. V. S. Rodrigues , M. B. Deroide , W. M. Takeshita , V. G. Garcia , R. S. de Molon , and L. H. Theodoro , “Efficacy of Antimicrobial Photodynamic Therapy for Treating Moderate to Deep Periodontal Pockets in Individuals With Type 2 Diabetes Mellitus: A Systematic Review and Meta‐Analysis,” Dentistry Journal 13, no. 1 (2025): 21. 39851597 10.3390/dj13010021 PMC11763938

[165] L. H. Theodoro , J. V. S. Rodrigues , M. M. Claudio , et al., “Effect of Butyl Toluidine Blue‐Mediated Photodynamic Therapy on Periodontal Healing in Type 2 Diabetic Patients. A Randomized Clinical Trial,” Journal of Dentistry 163 (2025): 106060. 40865714 10.1016/j.jdent.2025.106060

[166] F. O. Costa , R. P. Esteves Lima , A. M. Costa , et al., “Adjunctive Effects of Photodynamic Therapy Using Indocyanine Green in Residual Pockets During Periodontal Maintenance Therapy: A Split‐Mouth Randomized Controlled Trial,” Journal of Periodontology 94, no. 9 (2023): 1100–1111. 37051740 10.1002/JPER.22-0672

[167] P. H. Petrilli , J. V. S. Rodrigues , M. J. P. Santos , et al., “Adjunctive Methylene Blue‐Based Antimicrobial Photodynamic Therapy in the Re‐Instrumentation of Residual Periodontal Pockets in Individuals With Down Syndrome: A Randomized Controlled Clinical Trial,” Journal of Dentistry 162 (2025): 106061. 40865715 10.1016/j.jdent.2025.106061

[168] M. Annunziata , G. Donnarumma , A. Guida , et al., “Clinical and Microbiological Efficacy of Indocyanine Green‐Based Antimicrobial Photodynamic Therapy as an Adjunct to Non‐Surgical Treatment of Periodontitis: A Randomized Controlled Clinical Trial,” Clinical Oral Investigations 27, no. 5 (2023): 2385–2394. 36719506 10.1007/s00784-023-04875-w PMC10159973

[169] C. T. Kassa , L. T. C. Salviatto , A. Tortamano , et al., “Antimicrobial Photodynamic Therapy Mediated by Methylene Blue in Surfactant Vehicle as Adjuvant to Periodontal Treatment. Randomized, Controlled, Double‐Blind Clinical Trial,” Photodiagnosis and Photodynamic Therapy 41 (2023): 103194. 36402375 10.1016/j.pdpdt.2022.103194

[170] R. Wiench , J. Fiegler‐Rudol , K. Grzech‐Lesniak , D. Skaba , and J. Arnabat‐Dominguez , “Photodithazine‐Mediated Antimicrobial Photodynamic Therapy: A Systematic Review of Efficacy and Applications,” International Journal of Molecular Sciences 26, no. 16 (2025): 8049. 40869370 10.3390/ijms26168049 PMC12386792

[171] R. Wiench , J. Fiegler‐Rudol , K. Latusek , et al., “Indocyanine Green as a Photosensitizer in Periodontitis Treatment: A Systematic Review of Randomized Controlled Trials,” Life (Basel) 15, no. 7 (2025): 1015. 40724518 10.3390/life15071015 PMC12300888

[172] J. M. Goodson , A. C. Tanner , A. D. Haffajee , G. C. Sornberger , and S. S. Socransky , “Patterns of Progression and Regression of Advanced Destructive Periodontal Disease,” Journal of Clinical Periodontology 9, no. 6 (1982): 472–481. 6960023 10.1111/j.1600-051x.1982.tb02108.x

[173] M. Nakajima , M. Yanagawa , H. Takikawa , et al., “Advances in Local Drug Delivery for Periodontal Treatment: Present Strategies and Future Directions,” Biomolecules 15, no. 6 (2025): 903. 40563543 10.3390/biom15060903 PMC12191426

[174] C. A. Ramseier , J. S. Kinney , A. E. Herr , et al., “Identification of Pathogen and Host‐Response Markers Correlated With Periodontal Disease,” Journal of Periodontology 80, no. 3 (2009): 436–446. 19254128 10.1902/jop.2009.080480 PMC5695217

[175] M. Witt , M. Cherri , M. Ferraro , et al., “Anti‐Inflammatory IL‐8 Regulation via an Advanced Drug Delivery System at the Oral Mucosa,” ACS Applied Bio Materials 6, no. 6 (2023): 2145–2157. 10.1021/acsabm.3c00024 37216981

[176] K. Rajes , P. Nolte , C. V. Yapto , K. Danker , H. Dommisch , and R. Haag , “Novel Adhesive Nanocarriers Based on Mussel‐Inspired Polyglycerols for the Application Onto Mucosal Tissues,” Pharmaceutics 14, no. 5 (2022): 940. 35631526 10.3390/pharmaceutics14050940 PMC9144514

[177] H. Dommisch , K. N. Stolte , J. Jager , et al., “Characterization of an Ester‐Based Core‐Multishell (CMS) Nanocarrier for the Topical Application at the Oral Mucosa,” Clinical Oral Investigations 25, no. 10 (2021): 5795–5805. 33821321 10.1007/s00784-021-03884-x PMC8443517

[178] J. Jager , K. Obst , S. B. Lohan , et al., “Characterization of Hyperbranched Core‐Multishell Nanocarriers as an Innovative Drug Delivery System for the Application at the Oral Mucosa,” Journal of Periodontal Research 53, no. 1 (2018): 57–65. 28898420 10.1111/jre.12487

[179] C. V. Yapto , K. Rajes , A. Inselmann , et al., “Topical Application of Dexamethasone‐Loaded Core‐Multishell Nanocarriers Against Oral Mucosal Inflammation,” Macromolecular Bioscience 24, no. 12 (2024): e2400286. 39363619 10.1002/mabi.202400286 PMC11648588

[180] P. Lu , D. Ruan , M. Huang , et al., “Harnessing the Potential of Hydrogels for Advanced Therapeutic Applications: Current Achievements and Future Directions,” Signal Transduction and Targeted Therapy 9, no. 1 (2024): 166. 38945949 10.1038/s41392-024-01852-x PMC11214942

[181] Z. Sha , Y. Wu , Y. Zheng , et al., “Advances in pH‐Responsive Drug Delivery Systems for Periodontitis Treatment,” Drug Delivery 32, no. 1 (2025): 2522109. 40574628 10.1080/10717544.2025.2522109 PMC12203708

[182] Y. Zhu , Z. Xiu , X. Jiang , et al., “Injectable Hydrogels With ROS‐Triggered Drug Release Enable the Co‐Delivery of Antibacterial Agent and Anti‐Inflammatory Nanoparticle for Periodontitis Treatment,” Journal of Nanobiotechnology 23, no. 1 (2025): 205. 40075491 10.1186/s12951-025-03275-4 PMC11900060

[183] C. S. Miller , C. P. King, Jr. , M. C. Langub , R. J. Kryscio , and M. V. Thomas , “Salivary Biomarkers of Existing Periodontal Disease: A Cross‐Sectional Study,” Journal of the American Dental Association (1939) 137, no. 3 (2006): 322–329. 16570465 10.14219/jada.archive.2006.0181

[184] F. E. Freeman , P. Pitacco , L. H. A. Van Dommelen , et al., “Development of a 3D Bioprinted Scaffold With Spatio‐Temporally Defined Patterns of BMP‐2 and VEGF for the Regeneration of Large Bone Defects,” Bio‐Protocol 11, no. 21 (2021): e4219. 34859133 10.21769/BioProtoc.4219 PMC8595425

[185] Z. Liu , Z. Xu , X. Wang , et al., “Construction and Osteogenic Effects of 3D‐Printed Porous Titanium Alloy Loaded With VEGF/BMP‐2 Shell‐Core Microspheres in a Sustained‐Release System,” Frontiers in Bioengineering and Biotechnology 10 (2022): 1028278. 36338136 10.3389/fbioe.2022.1028278 PMC9634119

[186] T. M. Figueiredo , G. do Amaral , G. N. Bezerra , L. Y. S. Nakao , and C. C. Villar , “Three‐Dimensional‐Printed Scaffolds for Periodontal Regeneration: A Systematic Review,” Journal of the Indian Society of Periodontology 27, no. 5 (2023): 451–460. 10.4103/jisp.jisp_350_22 PMC10538520 37781321

[187] J. Y. Park , J. H. Shim , S. A. Choi , et al., “3D Printing Technology to Control BMP‐2 and VEGF Delivery Spatially and Temporally to Promote Large‐Volume Bone Regeneration,” Journal of Materials Chemistry B 3, no. 27 (2015): 5415–5425. 32262513 10.1039/c5tb00637f

[188] A. J. Bonito , L. Lux , and K. N. Lohr , “Impact of Local Adjuncts to Scaling and Root Planing in Periodontal Disease Therapy: A Systematic Review,” Journal of Periodontology 76, no. 8 (2005): 1227–1236. 16101353 10.1902/jop.2005.76.8.1227

[189] W. A. Soskolne , “Subgingival Delivery of Therapeutic Agents in the Treatment of Periodontal Diseases,” Critical Reviews in Oral Biology and Medicine 8, no. 2 (1997): 164–174. 9167091 10.1177/10454411970080020501

[190] B. Yin , J. M. Dodda , S. H. D. Wong , et al., “Smart Injectable Hydrogels for Periodontal Regeneration: Recent Advancements in Biomaterials and Biofabrication Strategies,” Materials Today Bio 32 (2025): 101855. 10.1016/j.mtbio.2025.101855 PMC12145717 40487163

[191] N. Castro Dos Santos , A. Mangussi , T. Ribeiro , et al., “Factors Influencing the Response to Periodontal Therapy in Patients With Diabetes: Post Hoc Analysis of a Randomized Clinical Trial Using Machine Learning,” Journal of Applied Oral Science 33 (2025): e20250211. 40736087 10.1590/1678-7757-2025-0211 PMC12652437

[192] C. P. Furquim , L. Caruth , G. Chandrasekaran , et al., “Developing Predictive Models for Periodontitis Progression Using Artificial Intelligence: A Longitudinal Cohort Study,” Journal of Clinical Periodontology 52 (2025): 1478–1490. 40830987 10.1111/jcpe.14194 PMC12420084

[193] E. Uzar , I. Pence , M. S. Cesmeli , and Z. Yetkin Ay , “Classification Success of Salivary Interleukin‐1beta in Periodontitis Grading With Artificial Intelligence Models: A Cross‐Sectional Observational Study,” Journal of Applied Oral Science 33 (2025): e20240580. 40802302 10.1590/1678-7757-2024-0580 PMC12652431

[194] W. V. Giannobile , “Artificial Intelligence to Advance Precision Oral Health,” International Journal of Periodontics & Restorative Dentistry 45, no. 5 (2025): 436–437. 40644336 10.11607/prd.7762

[195] L. R. Tao , Y. Li , X. Y. Wu , et al., “Deep Learning Photo Processing for Periodontitis Screening,” Journal of Dental Research (2025): 220345251347508, 10.1177/00220345251347508. 40650464

[196] R. S. de Molon , “From Promise to Practice: Can AI Apps Really Monitor Oral Health at Home?,” British Dental Journal 239, no. 5 (2025): 321. 10.1038/s41415-025-9163-8 40940476

[197] D. F. Kinane , H. Shiba , and T. C. Hart , “The Genetic Basis of Periodontitis,” Periodontology 2000 39 (2005): 91–117. 16135066 10.1111/j.1600-0757.2005.00118.x

[198] M. Gundelly , S. V. Pusuluri , R. R. Koduganti , M. Ambati , S. Chiluveru , and M. Chandaka , “Precision Medicine in Periodontics: A Literature Review,” Cureus 16, no. 9 (2024): e68952. 39385855 10.7759/cureus.68952 PMC11461172

[199] J. Korgaonkar , A. Y. Tarman , H. Ceylan Koydemir , and S. S. Chukkapalli , “Periodontal Disease and Emerging Point‐Of‐Care Technologies for Its Diagnosis,” Lab on a Chip 24, no. 14 (2024): 3326–3346. 38874483 10.1039/d4lc00295d

[200] M. Feres , B. Retamal‐Valdes , M. Faveri , et al., “Proposal of a Clinical Endpoint for Periodontal Trials: The Treat‐To‐Target Approach,” Journal of the International Academy of Periodontology 22, no. 2 (2020): 41–53. 32224549

[201] T. Kikuchi , J. I. Hayashi , and A. Mitani , “Next‐Generation Examination, Diagnosis, and Personalized Medicine in Periodontal Disease,” Journal of Personalized Medicine 12, no. 10 (2022): 1743. 36294882 10.3390/jpm12101743 PMC9605396

[202] M. Adelfio , Z. Martin‐Moldes , J. Erndt‐Marino , et al., “Three‐Dimensional Humanized Model of the Periodontal Gingival Pocket to Study Oral Microbiome,” Advanced Science 10, no. 12 (2023): e2205473. 36825685 10.1002/advs.202205473 PMC10131835

[203] S. Fan , Y. Ge , B. Li , P. Liu , and X. Liu , “Advancements in Microfluidic Organ‐On‐a‐Chip for Oral Medicine,” International Dental Journal 75, no. 5 (2025): 100925. 40743817 10.1016/j.identj.2025.100925 PMC12329541

[204] H. Makkar and G. Sriram , “Advances in Modeling Periodontal Host‐Microbe Interactions: Insights From Organotypic and Organ‐On‐Chip Systems,” Lab on a Chip 25, no. 5 (2025): 1342–1371. 39963082 10.1039/d4lc00871e PMC11833442

[205] eBioMedicine , “The 3Rs of Animal Research,” eBioMedicine 76 (2022): 103900. 35221013 10.1016/j.ebiom.2022.103900 PMC8882996

[206] R. C. Hubrecht and E. Carter , “The 3Rs aCnd Humane Experimental Technique: Implementing Change,” Animals (Basel) 9, no. 10 (2019): 754. 31575048 10.3390/ani9100754 PMC6826930

[207] W. T. Poh and J. Stanslas , “The New Paradigm in Animal Testing – 3Rs Alternatives,” Regulatory Toxicology and Pharmacology 153 (2024): 105705. 39299677 10.1016/j.yrtph.2024.105705

